# Genome-wide mapping of Quantitative Trait Loci for fatness, fat cell characteristics and fat metabolism in three porcine F_2 _crosses

**DOI:** 10.1186/1297-9686-42-31

**Published:** 2010-07-28

**Authors:** Hermann Geldermann, Stanislav Čepica, Antonin Stratil, Heinz Bartenschlager, Siegfried Preuss

**Affiliations:** 1 Animal Breeding and Biotechnology, University of Hohenheim, Stuttgart, Germany; 2 Institute of Animal Physiology and Genetics, Academy of Sciences of the Czech Republic, Liběchov, Czech Republic; 3 Department of Animal Breeding and Biotechnology, University of Hohenheim, Stuttgart, Germany

## Abstract

**Background:**

QTL affecting fat deposition related performance traits have been considered in several studies and mapped on numerous porcine chromosomes. However, activity of specific enzymes, protein content and cell structure in fat tissue probably depend on a smaller number of genes than traits related to fat content in carcass. Thus, in this work traits related to metabolic and cytological features of back fat tissue and fat related performance traits were investigated in a genome-wide QTL analysis. QTL similarities and differences were examined between three F_2 _crosses, and between male and female animals.

**Methods:**

A total of 966 F_2 _animals originating from crosses between Meishan (M), Pietrain (P) and European wild boar (W) were analysed for traits related to fat performance (11), enzymatic activity (9) and number and volume of fat cells (20). Per cross, 216 (M × P), 169 (W × P) and 195 (W × M) genome-wide distributed marker loci were genotyped. QTL mapping was performed separately for each cross in steps of 1 cM and steps were reduced when the distance between loci was shorter. The additive and dominant components of QTL positions were detected stepwise by using a multiple position model.

**Results:**

A total of 147 genome-wide significant QTL (76 at P < 0.05 and 71 at P < 0.01) were detected for the three crosses. Most of the QTL were identified on SSC1 (between 76-78 and 87-90 cM), SSC7 (predominantly in the MHC region) and SSCX (in the vicinity of the gene *CAPN6*). Additional genome-wide significant QTL were found on SSC8, 12, 13, 14, 16, and 18. In many cases, the QTL are mainly additive and differ between F_2 _crosses. Many of the QTL profiles possess multiple peaks especially in regions with a high marker density. Sex specific analyses, performed for example on SSC6, SSC7 and SSCX, show that for some traits the positions differ between male and female animals. For the selected traits, the additive and dominant components that were analysed for QTL positions on different chromosomes, explain in combination up to 23% of the total trait variance.

**Conclusions:**

Our results reveal specific and partly new QTL positions across genetically diverse pig crosses. For some of the traits associated with specific enzymes, protein content and cell structure in fat tissue, it is the first time that they are included in a QTL analysis. They provide large-scale information to analyse causative genes and useful data for the pig industry.

## Background

Reduced fatness improves carcass value, and therefore numerous studies on QTL mapping in pig concern fat deposition related traits (see reviews [1,2]). More recently, the results have been compiled in the database PigQTLdb ([3,4]; http://www.animalgenome.org/QTLdb/pig.html). As shown in several studies, QTL profiles depend largely on genetic resources, trait definition and statistical models. Taken together, these studies have detected major QTL affecting fat traits on porcine chromosomes SSC1, 2, 4, 6, 7 and X.

Traits like volume of adipose tissue and fat metabolism are influenced by lipogenesis and lipolysis rates, relationship between lipogenesis and lipolysis, energy intake and adipocyte differentiation. In pig, fat accretion is related to the activity of NADPH-generating enzymes in adipose tissue [5]. Strutz [6] has reported genetic correlations of about -0.4 to -0.6 between carcass fat content and activity of NADPH-generating enzymes. The content of soluble proteins in porcine fat tissue is an indicator of metabolic activity and has been reported to be genetically correlated (about -0.5) with fat content in carcass [7]. Furthermore, data on the diameter and number of porcine fat cells and on cell size differences between lean and obese pigs have been reported [8,9].

Activity of specific enzymes, protein content and cell structure in fat tissue probably depend on a smaller number of genes than production traits related to fat content in carcass. Thus, we have measured metabolic and cytological features for back fat tissue together with performance traits related to carcass fat deposition and used these traits in a genome-wide QTL analysis.

The positions of the QTL were compared among three F_2 _porcine crosses as well as between male and female animals. For some traits, we analysed the combined influence of QTL positioned on different chromosomes on the trait variance. We detected a total of 76 QTL (P < 0.05) and 71 QTL (P < 0.01) with genome-wide significant effects for the three crosses, but numerous QTL were observed only in one or two of the crosses.

## Methods

### Animals

A total of 966 F_2 _pigs were generated with founder animals from the Meishan and Pietrain breeds and the European wild boar (Table 1). All pigs were maintained under standardized housing in one experimental station. Generation of animals for the three F_2 _crosses and conditions of feeding are described elsewhere [2,10].

**Table 1:** Pedigrees of the three F_2 _crosses with animal numbers used in the calculations

Generation	Number of animals								
	♂M × ♀P			♂W × ♀P			♂W × ♀M		
			
	♂	♀	Σ	♂	♀	Σ	♂	♀	Σ
Founder	1	8	9	1	9	10	1	4	5
F_1_	3	19	22	2	26	28	2	21	23
F_2_	170	146	316	150	165	315	169	166	335

M: Meishan; P: Pietrain; W: European wild boar

### Sampling

Blood samples were collected from founders, F_1 _and F_2 _animals. Blood was taken from the *v. jugularis *of living animals or during stunning and separated into plasma, erythrocytes and leucocytes. DNA was isolated from the leukocyte fraction by chloroform-phenol extraction according to standard protocols.

Adipose tissue of the back fat area between the skin and *m. longissimus dorsi *at 13^th^/14^th ^rib was collected from the F_2 _animals directly after stunning. For each animal, a piece of back fat tissue was sampled and stored immediately in liquid nitrogen. After thawing the subcutaneous adipose tissue at the connective tissue border was separated into an inner and outer layer sample. For both samples, connective tissue and blood vessels were removed and the adipose tissue used immediately.

### Trait measurements

As shown in Table 2, 40 traits were recorded, including 11 performance traits associated with fat deposition (Table 2a). Six other traits related to enzyme activities and three to protein content were measured in fat tissue (Table 2b). The relative numbers or volumes of fat cells were determined using different parameters defining 20 traits (Table 2c). Traits related to protein content, enzyme activities and fat cells are described in the following sections.

**Table 2:** Definition of traits^a^

**a) **Performance traits associated with fatness		
Acronym	Definition	Unit

CW	Carcass weight (weight of carcass with kidneys, 24 h after slaughter, cold)	kg
AFW	Abdominal fat weight	kg
HEFW	Ham external fat weight	kg
SEFW	Shoulder external fat weight	kg
BFW	Back fat weight (loin and neck external fat weight)	kg
FCP	Fat cuts (weight of external fat from ham, shoulder, loin, neck as well as abdominal fat, as proportion of carcass weight)	%
BFML	Back fat depth on *M. long. dorsi *at 13^th^/14^th ^rib (average of three measurements at three points, lateral to the cutting line of chops)	mm
FD10	Fat depth at 10^th ^rib (depth of fat and skin on muscle, average of three measurements, at thinnest point)	mm
ABFD	Average back fat depth (mean value of shoulder fat depth, fat depth at about 10^th ^rib and loin fat depth)	mm
FAML	Fat area on *M. long. dorsi *at 13^th^/14^th ^rib (back fat area according to [40])	cm^2^
FMR	Fat to meat ratio (fat area in relation to meat area at 13^th^/14^th ^rib)	

**b) **Enzyme activity and protein content measured from fat tissue		

Acronym	Definition	Unit

MDHO	Activity of NADP-malate dehydrogenase, outer back fat layer	units/g tissue
PCO	Protein content, outer back fat layer	mg/g tissue
LGSEO	Logarithm of activity of NADPH generating enzymes, outer back fat layer(transformed for normal distribution of the trait)	lg_10 _(units/g tissue * 1000)
MDHI	Activity of NADP-malate dehydrogenase, inner back fat layer	units/g tissue
PCI	Protein content, inner back fat layer	mg/g tissue
LGSEI	Logarithm of activity of NADPH generating enzymes, inner back fat layer(transformed for normal distribution of the trait)	lg_10 _(units/g tissue * 1000)
MDHOI	Activity of NADP-malate dehydrogenase, averaged outer and inner back fat layer	units/g tissue
PCOI	Protein content, averaged outer and inner back fat layer	mg/g tissue
LGSEOI	Logarithm of activity of NADPH generating enzymes (ICDH + MDH + 6PGDH + G6PDH), averaged outer and inner back fat layer (transformed for normal distribution of the trait)	lg_10 _(units/g tissue * 1000)

**c) **Relative numbers and volumes of fat cells with different diameters		

Acronym	Definition	Unit

FN73	Relative number of fat cells in the class of about 73 μm diameter	%
FN92	Relative number of fat cells in the class of about 92 μm diameter	%
FN114	Relative number of fat cells in the class of about 114 μm diameter	%
FN146	Relative number of fat cells in the class of about 146 μm diameter	%
FN183	Relative number of fat cells in the class of about 183 μm diameter	%
FNCM	Relative number of fat cells with medium cell sizes (FN73 + FN92 + FN114)	%
FNCL	Relative number of fat cells with large cell sizes (FN146 + FN183 + FN228).FN228 is not included as separate trait.	%
RFNCSL	Ratio of FNCS/FNCL (FN23 + FN29 + FN36 + FN41 + FN57)/(FN146 + FN183 + FN228). FNCS (small cell sizes) is not included as separate trait.	
RFNCML	Ratio of FNCM/FNCL (FN73 + FN92 + FN114)/(FN146 + FN183 + FN228)	
RFNCLO	Ratio of FNCL/(FNCS + FNCM)(FN146 + FN183 + FN228)/(FN23 + FN29 + ... + FN114)	
FV73	Relative volume of fat cells in the class of about 73 μm diameter	%
FV92	Relative volume of fat cells in the class of about 92 μm diameter	%
FV114	Relative volume of fat cells in the class of about 114 μm diameter	%
FV146	Relative volume of fat cells in the class of about 146 μm diameter	%
FV183	Relative volume of fat cells in the class of about 183 μm diameter	%
FVCM	Relative volume of fat cells with medium cell sizes (FV73 + FV92 + FV114)	%
FVCL	Relative volume of fat cells with large cell sizes (FV146 + FV183 + FV228).FV228 is not included as separate trait.	%
RFVCSL	Ratio of FVCS/FVCL (FV23 + FV29 + ... + FV57)/(FV146 + FV183 + FV228). FVCS (small cell sizes) is not included as separate trait.	
RFVCML	Ratio of FVCM/FVCL(FV73 + FV92 + FV114)/(FV146 + FV183 + FV228)	
RFVCLO	Ratio of FVCL/(FVCS + FVCM)(FV146 + FV183 + FV228)/(FV23 + FV29 + ... + FV114)	

^a ^Data of Mean, SD, N and r^2 ^are given in Additional file 2

#### Soluble proteins and enzymes

Each fat tissue sample was cut into small pieces (about 1 mm thick) and then homogenized at 0°C in a 0.15 M KCl solution. The homogenate was centrifuged (20 min, 20000 g, +4°C) and the supernatant filtered (Filter No. 11303, pore diameter 1.2 μm, Sartorius, Göttingen, Germany). The filtrate was kept at +4°C and immediately used to measure protein content and enzyme activities. Protein contents were estimated according to [11]. For each fat tissue sample, protein content was measured three times and averaged. To measure each enzyme activity, 0.1 mL of the filtrate was mixed:

- for isocitrate dehydrogenase (ICDH): with 1.0 mL of 0.075 M glycyl-glycine buffer (pH 7.4), 0.1 mL of 0.05 M MnCl_2_-4H_2_O, 0.2 mL of 0.002 M NADP, 1.5 mL H_2_O, and 0.1 mL of 0.06 M 1.5 DL-isocitrate;

- for malate dehydrogenase (MDH): with 2.0 mL of 0.3 M Tris/HCl buffer (pH 8.5), 0.6 mL of 0.01 M MnSO_4_-H_2_O, 0.6 mL of 0.002 M NADP, 2.1 mL H_2_O, and 0.6 mL of 1 M malate;

- for 6-phosphogluconate dehydrogenase (6PGDH) and glucose-6-phosphate dehydrogenase (G6PDH): with 0.5 mL of 0.25 M glycyl-glycine buffer (pH 8.0), 0.5 mL of 0.2 M MgCl_2_-6H_2_O, 0.2 mL of 0.0075 M NADP, 0.8 mL H_2_O, 0.3 mL of 0.01 M 6-phosphogluconate (6PG), and 0.01 M glucose-6-phosphate (G6P).

The mixtures were incubated for 3 min at 30°C, and the absorbance was measured at 340 nm with a photometer (Perkin Elmer, Wellesley, MA, USA) for 5 min. The activity was calculated in IU per g of tissue. For each fat tissue sample, enzyme activities were measured twice and averaged. For further details on protein and enzyme traits see Table 2b.

#### Fat cell traits

According to the methods described in [12-14], each fat tissue sample was cut up with minimal pressure into slices about 1 mm thick. One g of tissue was suspended in 3 mL KRB buffer (Krebs-Ringer bicarbonate buffer with 5 mM glucose and 25 mM HEPES, pH 7.4) containing 3 mg/mL collagenase and slowly stirred at 37°C for 1 h.

The prepared cell suspension was filtered (PP filter, 1000 μm, Sartorius, Göttingen, Germany), collected in 3 mL KRB buffer, sedimented and again suspended in 3 mL KRB buffer. Then, 500 μL cell suspension were incubated with 5 mL collidine-HCl buffer (1 M 2,4,6-trimethylpyridine, 0.1 M HCl, 0.26 M NaCl, pH 7.4) and 3 mL OsO_4 _solution (3% w/v OsO_4 _in collidine-HCl buffer) for 24 h at room temperature. The number of suspended cells was measured with a Coulter-Counter (Model TA II, Beckman, Krefeld, Germany) in different size fractions. In practise, the particle counter measured the changes of resistance caused by individual particles passing the opening of a capillary wall with electrodes on both sides. Using an automatic coincidence correction guarantied that particles passing simultaneously were counted separately. Assuming spherical particles, the particle numbers and volumes were calculated for size classes with cell diameters of 23, 29, 36, 41, 57, 73, 92, 114, 146, 183, and 228 μm.

### Marker loci and genotyping

Marker loci were selected to be informative, evenly distributed over the chromosomes, and nearly the same for the three crosses. Only when the information content of a selected locus within a cross was low, was an alternative flanking locus chosen for that cross. For regions with previously detected QTL for performance traits [2] on SSC2, SSC4 and SSCX, high marker density maps were built. Per cross, 216 (M × P), 169 (W × P) and 195 (W × M) polymorphic markers were genotyped (Table 3). Marker loci parameters (map position, number of alleles, observed informative meioses etc.) and polymorphism types are provided in Additional file 1.

**Table 3:** Overview of marker loci and chromosomes^a^

Parameter	M × P	W × P	W × M
Number of marker loci			
Total	216	169	195
Microsatellites	138	131	138
SNPs	56	18	38
Other polymorphisms^b^	22	20	19

Number of markers per chromosome			
Average	11.4	8.9	10.3
Min.	4	3	3
Max.	29	17	20

Total map size^c^	2762	2692	2728

Map size per chromosome^c^			
Average	145.4	141.7	143.6
Min.	56.4	48.7	58.8
Max.	232.1	229.2	235.9

Average marker interval^c^	14.0	17.9	15.5

^a ^additional information on marker loci is provided in Additional file 1; ^b^allotypes, blood groups, biochemical polymorphisms, indels, SSCPs, DGGEs; ^c ^sex averaged lengths/intervals for the loci in Kosambi cM for the F_2 _crosses

### Statistical analyses

#### Linkage mapping of marker loci and calculation of trait values

Linkage mapping was performed using the CriMap software, version 2.4 [15,16]. The information content of each locus for mapping was assessed by the number of informative meioses (Additional file 1). The number of informative meioses averaged across all loci was 558 (702) for the M × P cross, 520 (722) for the W × P cross and 623 (732) for the W × M cross, the number in brackets being the maximum number of informative meioses for a locus. The frequencies of the observed informative meioses per cross were 0.79 (M × P), 0.72 (W × P) and 0.85 (W × M).

Additional file 2 contains the numbers of observations, phenotypic means, standard deviations and determination coefficients of the traits for the F_2 _animals of each cross.

#### QTL analysis

The least square method was applied for QTL mapping [17] and was performed separately for each of the three crosses in steps of 1 cM; the steps were reduced when the distance between marker loci was shorter. As described for the autosomes in [3] and for chromosome X in [18], the conditional probabilities for the transfer of an allele from the founder to the F_2 _individual were calculated for any position of the linkage array by considering all marker loci of a linkage group simultaneously and stored as additive and dominant components. From these linear components, the additive and dominant effects were calculated for each trait in a generalized linear model procedure (GLM) including the continuous (age at slaughter) and discontinuous (two-month classes of seasonal influence, sex, litter number) independent variables. Only 91 W × M F_2 _animals were measured for fat cell traits, which were not adjusted for the effects of season and litter number in our models because of insufficient connectedness of these independent variables. The mean square estimates of the additive and dominant components in relation to the error variance was calculated from the complete model, and the position on a chromosome with the highest *F *ratio value was considered as the most likely QTL position. Genome-wide (P < 0.05) significant QTL maxima (major peaks) were determined for all traits (Table 4). Additional genome-wide significant minor peaks were registered per trait and chromosome with P < 0.01 for performance traits (Table 2a) and P < 0.05 for the other traits (Table 2b and 2c) when they were more than 20 cM away from the major peak and from the already considered minor peaks.

**Table 4:** Genome-wide significant QTL for fat related traits identified in the three Hohenheim crosses

SSC	Trait^a^	Cross^b^	Position^c^		Flanking markers^d^	*F *ratio^e^		VF_2_^f^	a ± SE^g^	d ± SE^g^
			USDA	Hoh.	**proximal/distal**					
1	CW	W × M	54.1	69.0	*SW2130/IGFR*	10.0	*	5.3	-4.44 ± 1.00	1.35 ± 1.64
	CW	W × P	77.4	115.7	*SW307/S0082*	15.9	**	8.9	-6.12 ± 1.09	0.65 ± 1.55
	CW	W × P	44.8	62.7	*S0008/SW2130*	14.5	**	8.2	-5.73 ± 1.07	-0.36 ± 1.68
	CW	W × P	59.1	87.9	*SW2130/SW307*	13.1	**	7.4	-6.15 ± 1.21	0.08 ± 2.05
	AFW	M × P	142.7	207.2	*EAA*	8.5	*	4.6	0.20 ± 0.05	-0.27 ± 0.11
	AFW	W × M	107.6	131.1	*TGFBR1/SW705*	10.3	*	5.4	-0.13 ± 0.03	0.13 ± 0.06
	AFW	W × P	76.3	112.7	*SW307/S0082*	9.2	*	5.1	-0.09 ± 0.02	0.06 ± 0.03
	HEFW	W × M	57.2	73.0	*SW2130/IGFR*	13.6	**	7.2	-0.30 ± 0.06	0.03 ± 0.10
	HEFW	W × M	91.5	114.7	*TPM2*	10.7	**	5.6	-0.17 ± 0.05	0.21 ± 0.07
	HEFW	W × P	77.8	116.7	*SW307/S0082*	20.7	**	11.5	-0.26 ± 0.04	0.04 ± 0.06
	HEFW	W × P	45.4	64.7	*S0008/SW2130*	10.6	**	5.9	-0.19 ± 0.04	-0.03 ± 0.07
	HEFW	W × P	64.4	93.9	*SW2130/SW307*	12.9	**	7.2	-0.23 ± 0.05	-0.01 ± 0.07
	SEFW	W × M	113.4	137.1	*TGFBR1/SW705*	11.0	**	5.8	-0.12 ± 0.03	0.01 ± 0.04
	SEFW	W × P	86.9	136.3	*SW780/SW803*	11.1	**	6.2	-0.10 ± 0.02	0.00 ± 0.04
	SEFW	W × P	76.7	113.7	*SW307/S0082*	11.0	**	6.2	-0.10 ± 0.02	0.00 ± 0.03
	BFW	W × M	107.6	131.1	*TGFBR1/SW705*	15.5	**	8.2	-0.33 ± 0.07	0.29 ± 0.11
	BFW	W × P	77.1	114.7	*SW307/S0082*	19.6	**	10.9	-0.33 ± 0.05	0.08 ± 0.08
	BFW	W × P	63.5	92.9	*SW2130/SW307*	14.0	**	7.9	-0.31 ± 0.06	0.04 ± 0.10
	BFW	W × P	100.8	161.2	*SW803/SW705*	11.3	**	6.4	-0.28 ± 0.06	-0.05 ± 0.12
	FCP	M × P	139.3	201.3	*SW705/EAA*	9.3	*	5.1	1.92 ± 0.44	-0.77 ± 0.93
	FCP	W × M	104.7	128.1	*TGFBR1/SW705*	14.9	**	7.9	-1.41 ± 0.28	1.12 ± 0.46
	FCP	W × P	86.9	136.3	*SW780/SW803*	9.4	*	5.2	-1.14 ± 0.26	0.21 ± 0.45
	BFML	W × P	89.0	140.3	*SW780/SW803*	15.3	**	8.6	-2.58 ± 0.47	-0.26 ± 0.82
	BFML	W × P	76.3	112.7	*SW307/S0082*	14.8	**	8.3	-2.53 ± 0.47	0.29 ± 0.69
	FD10	W × M	89.5	112.9	*TPM2/SW803*	12.7	**	6.7	-2.20 ± 0.49	1.76 ± 0.73
	FD10	W × P	77.8	116.7	*SW307/S0082*	14.1	**	7.9	-2.11 ± 0.40	0.56 ± 0.56
	ABFD	W × M	91.4	114.6	*SW780/TPM2*	11.6	**	6.2	-1.90 ± 0.47	1.89 ± 0.68
	ABFD	W × P	78.4	118.2	*S0082/SW780*	12.5	**	7.0	-2.01 ± 0.40	0.44 ± 0.57
	FAML	W × P	76.7	113.7	*SW307/S0082*	15.6	**	8.8	-2.53 ± 0.46	0.25 ± 0.67
	FAML	W × P	87.4	137.3	*SW780/SW803*	15.0	**	8.5	-2.61 ± 0.48	0.27 ± 0.82
	FMR	M × P	113.8	166.3	*TGFBR1/SW705*	13.4	**	7.5	0.09 ± 0.02	0.04 ± 0.03
	FMR	M × P	135.8	196.3	*SW705/EAA*	12.1	**	6.8	0.11 ± 0.02	0.01 ± 0.05
	FMR	W × M	111.4	135.1	*TGFBR1/SW705*	8.8	*	4.6	-0.12 ± 0.03	0.01 ± 0.05
	FMR	W × P	90.6	143.3	*SW780/SW803*	12.8	**	7.3	-0.06 ± 0.01	-0.01 ± 0.02
	FMR	W × P	76.0	111.7	*SW307/S0082*	10.6	**	6.0	-0.06 ± 0.01	0.01 ± 0.02
	MDHO	W × P	94.3	150.2	*SW803*	9.1	*	5.1	-0.03 ± 0.01	-0.05 ± 0.02
	FV114	W × P	103.8	166.2	*SW803/SW705*	8.8	*	5.2	-4.93 ± 1.21	4.26 ± 2.65
	FVCM	W × M	91.5	114.7	*TPM2*	9.9	*	16.8	8.97 ± 2.50	-9.29 ± 3.85
	FVCM	W × M	111.4	135.1	*TGFBR1/SW705*	8.5	*	14.5	12.33 ± 3.00	0.88 ± 5.35
	FVCL	W × M	114.3	138.1	*TGFBR1/SW705*	9.2	*	15.7	-13.53 ± 3.16	-0.07 ± 5.44
	FVCL	W × M	91.5	114.7	*TPM2*	8.9	*	15.3	-9.19 ± 2.70	9.56 ± 4.15

2	CW	W × P	74.4	94.3	*SW395/S0010*	8.7	*	4.8	-3.80 ± 1.01	-3.41 ± 1.61
	HEFW	W × P	57.4	69.3	*MYOD1*	10.9	**	6.1	-0.19 ± 0.04	-0.14 ± 0.08
	SEFW	W × P	71.4	90.3	*SW395/S0010*	8.6	*	4.8	-0.06 ± 0.02	-0.08 ± 0.03
	BFW	W × P	72.9	92.3	*SW395/S0010*	11.0	**	6.2	-0.20 ± 0.05	-0.24 ± 0.08
	FCP	M × P	48.0	61.4	*SW240/MLP*	8.6	*	4.7	1.14 ± 0.28	0.29 ± 0.46
	FMR	M × P	49.7	63.4	*SW240/MLP*	9.8	*	5.4	0.07 ± 0.02	0.04 ± 0.03

4	CW	M × P	71.2	65.0	*SW1089/V-ATPase*	10.0	*	5.5	-4.84 ± 1.10	1.27 ± 1.59
	SEFW	M × P	77.6	79.4	*ATP1A2*	9.5	*	5.2	-0.11 ± 0.03	0.04 ± 0.04
	BFW	M × P	37.0	37.9	*SW835/SWR73*	11.4	**	6.3	-0.30 ± 0.07	-0.19 ± 0.10
	RFNCLO	W × P	54.7	59.2	*SW2128/SW1073*	9.9	*	5.9	-15.18 ± 3.52	-4.26 ± 5.59
	FV73	W × P	74.4	76.8	*S0073*	9.7	*	5.7	-2.67 ± 0.61	0.28 ± 0.95
	FV146	W × P	74.4	76.8	*S0073*	10.6	**	6.3	4.31 ± 0.99	-2.10 ± 1.53
	FVCL	W × P	73.9	75.9	*V-ATPase/S0073*	8.6	*	5.1	4.15 ± 1.11	-3.00 ± 1.74
	RFVCSL	W × P	53.0	58.2	*SW2128/SW1073*	9.7	*	5.8	-0.15 ± 0.04	-0.05 ± 0.05

5	CW	W × M	94.4	81.5	*S0005/SW152*	8.7	*	4.6	3.76 ± 0.94	1.80 ± 1.42
	SEFW	W × M	85.7	73.0	*SW2/S0005*	10.1	*	5.3	0.10 ± 0.02	0.05 ± 0.04

6	AFW	M × P	75.6	97.8	*TGFB1*	13.5	**	7.5	0.15 ± 0.03	0.11 ± 0.04
	HEFW	M × P	75.6	97.8	*TGFB1*	12.2	**	6.8	0.29 ± 0.06	0.15 ± 0.09
	SEFW	M × P	75.6	97.8	*TGFB1*	11.8	**	6.6	0.12 ± 0.03	0.07 ± 0.04
	BFW	M × P	75.6	97.8	*TGFB1*	9.4	*	5.2	0.26 ± 0.07	0.17 ± 0.09
	FCP	M × P	75.6	96.9	*LIPE*	28.1	**	15.0	1.87 ± 0.27	0.91 ± 0.36
	FCP	W × P	76.5	81.4	*A1BG*	11.4	**	6.4	0.77 ± 0.21	1.00 ± 0.31
	BFML	M × P	75.6	97.8	*TGFB1*	14.5	**	8.1	2.52 ± 0.50	1.20 ± 0.67
	BFML	W × P	76.5	81.4	*A1BG*	9.1	*	5.0	1.11 ± 0.40	1.94 ± 0.57
	FD10	M × P	75.6	97.8	*TGFB1*	9.1	*	5.0	1.51 ± 0.48	1.75 ± 0.64
	ABFD	M × P	75.6	97.8	*TGFB1*	11.8	**	6.6	1.80 ± 0.50	2.12 ± 0.67
	FMR	M × P	75.6	96.9	*LIPE*	17.8	**	9.9	0.10 ± 0.02	-0.01 ± 0.02
	FMR	W × P	78.5	88.2	*EAH/NPPB*	10.8	**	6.1	0.04 ± 0.01	0.05 ± 0.02

7	CW	W × M	67.2	87.9	*TNFB/S0102*	9.5	*	5.0	-3.74 ± 0.91	1.59 ± 1.34
	AFW	M × P	72.6	88.1	*S0102/PSMA4*	17.4	**	9.7	-0.19 ± 0.04	-0.08 ± 0.05
	SEFW	W × M	64.9	85.9	*TNFB/S0102*	9.9	*	5.2	-0.10 ± 0.02	0.05 ± 0.03
	BFML	M × P	63.9	78.8	*TNFB/S0102*	11.0	**	6.1	-2.17 ± 0.54	-1.99 ± 0.79
	FD10	M × P	63.9	78.8	*TNFB/S0102*	23.8	**	12.9	-3.27 ± 0.49	-1.31 ± 0.71
	FD10	W × M	57.7	78.8	*TNFA*	16.9	**	8.9	2.73 ± 0.47	0.10 ± 0.67
	ABFD	M × P	60.4	75.8	*TNFB/S0102*	18.6	**	10.3	-2.99 ± 0.51	-1.41 ± 0.73
	ABFD	W × M	57.7	78.8	*TNFA*	8.5	*	4.4	1.94 ± 0.48	0.48 ± 0.68
	MDHO	M × P	53.8	67.3	*S0064/KE6*	15.2	**	8.5	-0.12 ± 0.02	-0.03 ± 0.03
	MDHO	W × M	50.1	71.8	*SWR1078/TNFA*	9.4	*	5.0	0.06 ± 0.01	0.02 ± 0.02
	PCO	M × P	51.1	63.1	*S0064/KE6*	10.5	*	5.8	0.57 ± 0.13	0.14 ± 0.19
	LGSEO	M × P	53.1	66.2	*S0064/KE6*	10.8	**	6.0	-0.07 ± 0.02	-0.01 ± 0.02
	LGSEO	W × M	55.5	76.8	*SWR1078/TNFA*	10.8	**	5.9	0.04 ± 0.01	0.02 ± 0.01
	MDHI	M × P	63.9	78.8	*TNFB/S0102*	12.0	**	6.7	-0.13 ± 0.03	-0.03 ± 0.04
	MDHI	M × P	47.8	58.1	*S0064/KE6*	10.0	*	5.5	-0.13 ± 0.03	-0.01 ± 0.05
	MDHOI	M × P	62.8	77.8	*TNFB/S0102*	15.4	**	8.6	-0.12 ± 0.02	-0.03 ± 0.03
	MDHOI	W × M	50.1	71.8	*SWR1078/TNFA*	10.1	*	5.5	0.07 ± 0.02	-0.00 ± 0.02
	LGSEOI	M × P	63.9	78.8	*TNFB/S0102*	11.3	**	6.3	-0.07 ± 0.01	0.00 ± 0.02
	LGSEOI	W × M	56.6	77.8	*SWR1078/TNFA*	8.6	*	4.6	0.03 ± 0.01	0.01 ± 0.01
	FN73	M × P	59.3	74.8	*TNFB/S0102*	9.7	*	5.5	2.43 ± 0.55	0.42 ± 0.77
	FN92	M × P	54.5	68.3	*S0064/KE6*	13.4	**	7.7	4.28 ± 0.83	1.00 ± 1.16
	FN92	M × P	82.2	103.3	*PSMA4/S0066*	8.7	*	4.9	3.32 ± 0.80	-0.33 ± 1.08
	FN92	W × M	32.9	55.9	*SWR1078*	8.6	*	14.8	-1.82 ± 1.03	-5.78 ± 1.49
	FN146	M × P	58.1	73.8	*TNFB*	9.8	*	5.6	-3.55 ± 0.89	-2.53 ± 1.22
	FN183	M × P	62.8	77.8	*TNFB/S0102*	10.2	*	5.8	-1.49 ± 0.38	-1.31 ± 0.55
	FNCM	M × P	60.4	75.8	*TNFB/S0102*	12.0	**	6.9	8.22 ± 1.68	1.69 ± 2.38
	FNCM	M × P	80.9	101.3	*PSMA4/S0066*	9.7	*	5.5	7.19 ± 1.64	-1.32 ± 2.25
	FNCM	W × M	32.9	55.9	*SWR1078*	10.3	*	17.5	-3.07 ± 2.40	-15.39 ± 3.47
	FNCL	M × P	59.3	74.8	*TNFB/S0102*	12.0	**	6.9	-5.11 ± 1.16	-3.88 ± 1.63
	FV73	W × M	82.8	118.5	*S0066*	9.0	*	15.4	-2.31 ± 0.63	-1.68 ± 0.84
	FV92	M × P	58.1	73.8	*TFNB*	15.0	**	8.6	4.49 ± 0.85	1.84 ± 1.16
	FV92	W × P	74.6	79.8	*S0102/PSMA4*	9.9	*	5.9	3.33 ± 0.86	-2.91 ± 1.34
	FV92	W × P	90.7	106.4	*S0066/S0115*	9.3	*	5.5	3.59 ± 1.00	-5.31 ± 1.98
	FV114	M × P	60.4	75.8	*TNFB/S0102*	12.0	**	6.8	4.41 ± 0.98	3.01 ± 1.39
	FV114	M × P	80.9	101.3	*PSMA4/S0066*	8.8	*	5.0	4.01 ± 0.96	-0.93 ± 1.32
	FV146	M × P	58.1	73.8	*TNFB*	9.9	*	5.6	-5.64 ± 1.30	-2.11 ± 1.79
	FV146	W × P	90.1	105.4	*S0066/S0115*	9.0	*	5.3	-3.95 ± 1.17	6.35 ± 2.27
	FV146	W × P	75.7	81.8	*S0102/PSMA4*	8.8	*	5.2	-3.37 ± 1.00	3.77 ± 1.54
	FV183	M × P	62.8	77.8	*TNFB/S0102*	15.9	**	9.1	-4.83 ± 0.94	-3.50 ± 1.36
	FVCM	M × P	59.3	74.8	*TNFB/S0102*	21.2	**	12.0	10.48 ± 1.69	5.21 ± 2.35
	FVCM	M × P	88.4	112.2	*S0066/S0115*	9.6	*	5.4	8.26 ± 1.89	-1.96 ± 2.99
	FVCM	W × P	91.4	107.4	*S0066/S0115*	9.8	*	5.8	3.67 ± 1.24	-8.87 ± 2.49
	FVCL	M × P	59.3	74.8	*TNFB/S0102*	19.1	**	10.8	-10.78 ± 1.83	-5.37 ± 2.56
	FVCL	W × P	90.7	106.4	*S0066/S0115*	10.1	*	6.0	-4.17 ± 1.30	8.75 ± 2.57
	FVCL	W × P	75.2	80.8	*S0102/PSMA4*	9.2	*	5.4	-3.75 ± 1.12	4.56 ± 1.74
	RFVCML	M × P	58.1	73.8	*TNFB*	11.3	**	6.5	0.66 ± 0.14	0.14 ± 0.19
	RFVCLO	M × P	58.1	73.8	*TNFB*	9.5	*	5.4	0.70 ± 0.16	0.11 ± 0.22
	RFVCLO	W × M	82.8	118.5	*S0066*	8.9	*	15.2	-1.00 ± 0.28	-0.75 ± 0.37

8	FN73	W × M	108.2	116.4	*SW16/SW61*	9.6	*	16.4	-1.29 ± 0.85	-5.33 ± 1.31
	FN92	W × M	107.5	114.4	*SW16/SW61*	17.1	**	26.8	-0.33 ± 1.17	-9.87 ± 1.70
	FNCM	W × M	107.8	115.4	*SW16/SW61*	17.1	**	26.8	0.13 ± 2.80	-24.41 ± 4.17

9	CW	W × P	142.5	193.2	*SW1349*	8.9	*	4.9	-3.00 ± 0.96	3.66 ± 1.42

12	PCO	M × P	113.1	109.3	*SWR1021*	8.7	*	4.8	-0.32 ± 0.12	-0.51 ± 0.17
	FV146	W × M	106.6	135.4	*S0106/SWR1021*	9.3	*	15.9	2.14 ± 2.69	16.69 ± 4.06

13	FN185	M × P	98.2	129.8	*SW520/SW38*	9.3	*	5.3	0.99 ± 0.37	-2.08 ± 0.55
	FV185	M × P	95.5	126.8	*SW520/SW38*	8.9	*	5.1	2.03 ± 1.00	-6.21 ± 1.56

14	RFNCSL	W × P	48.0	52.8	*SW210/SW2488*	9.7	*	5.8	10.19 ± 2.46	-4.25 ± 3.97
	RFNCML	W × P	56.2	62.8	*SW210/SW2488*	9.9	*	5.9	6.63 ± 1.71	-6.02 ± 3.06
	RFNCLO	W × P	51.3	56.8	*SW210/SW2488*	11.5	**	6.8	17.23 ± 3.83	-8.45 ± 6.57

16	FN73	M × P	34.1	43.0	*S0077/S0026*	8.5	*	4.8	-1.82 ± 0.54	-1.93 ± 0.76

18	PCO	M × P	19.0	25.6	*EAI/LEP*	9.6	*	5.3	-0.35 ± 0.13	0.64 ± 0.19

X	CW	W × M	80.0	90.0	*ACSL4/CAPN6*	10.0	*	10.3	-11.72 ± 2.89	7.36 ± 3.22
	HEFW	W × M	80.0	90.0	*ACSL4/CAPN6*	9.6	*	10.0	-0.58 ± 0.16	0.30 ± 0.18
	SEFW	W × P	80.6	105.5	*SW259/SW1943*	9.7	*	10.2	0.27 ± 0.08	-0.15 ± 0.09
	PCO	M × P	28.4	29.1	*SW980/SW2126*	9.4	*	10.8	3.22 ± 0.74	-3.25 ± 0.81
	PCO	M × P	122.2	139.3	*FMR1*	8.9	*	10.3	3.11 ± 0.74	-3.13 ± 0.78
	PCO	W × M	81.0	95.5	*CAPN6*	11.2	**	11.8	0.77 ± 0.26	-0.12 ± 0.28
	LGSEO	M × P	56.9	55.7	*SW2456/AR*	8.5	*	9.8	-0.38 ± 0.10	0.33 ± 0.10
	LGSEI	M × P	29.3	30.1	*SW980/SW2126*	11.4	**	13.1	-0.36 ± 0.08	0.38 ± 0.08
	LGSEI	M × P	113.8	128.2	*SW2453/FMR1*	11.3	**	13.1	-0.45 ± 0.09	0.43 ± 0.10
	PCI	W × M	80.4	92.0	*ACSL4/CAPN6*	11.0	*	11.7	0.83 ± 0.32	0.05 ± 0.36
	PCOI	W × M	80.7	94.0	*ACSL4/CAPN6*	12.5	**	13.1	0.80 ± 0.27	-0.04 ± 0.30
	LGSEOI	M × P	111.5	125.2	*SW2453/FMR1*	11.2	**	13.0	-0.46 ± 0.10	0.44 ± 0.11
	LGSEOI	M × P	29.3	30.1	*SW980/SW2126*	11.0	*	12.8	-0.34 ± 0.07	0.36 ± 0.08
	LGSEOI	M × P	56.9	55.7	*SW2456/AR*	11.2	**	12.9	-0.40 ± 0.09	0.37 ± 0.09
	FV73	M × P	55.4	53.7	*SW2456*	9.2	*	10.7	10.51 ± 2.75	-8.17 ± 2.84
	RFVCSL	M × P	55.4	53.7	*SW2456*	12.2	**	14.1	0.68 ± 0.15	-0.55 ± 0.15
	RFVCSL	M × P	126.0	144.2	*FMR1/SW2588*	10.9	*	12.7	0.67 ± 0.15	-0.57 ± 0.16

^a ^trait: acronym, for definition see Table 2; ^b ^cross: Hohenheim F_2 _crosses (M: Meishan, P: Pietrain, W: European wild boar); ^c ^position: USDA, position in USDA MARC map; Hoh.: position in Hohenheim map; ^d ^flanking markers: nearest proximal/distal locus in the Hohenheim map; if the QTL position coincides with that of the marker, only one locus is indicated; ^e ^*F *ratio: mean square estimates of the additive and dominant components in relation to the error variance of the model; significance for the genome wide 5% (*) and 1% (**) level calculated by permutation test [19]; for SSCX, only the results for female animals are listed; for threshold values see Table 5; ^f ^VF_2_: proportion of error variance reduction by inclusion of additive and dominant components in the initial model; ^g ^a: additive effect (positive/negative signs indicate the superior/inferior trait values inherited from the paternal resource group); d:dominant effect (positive for higher values of heterozygous individuals than the mean of homozygotes, negative for lower values); SE: standard error of estimates

For chromosomes SSC6, 7 and X, we performed separate calculations for female and male animals in order to test sex-specific differences in QTL positions and genetic effects. The model for these data sets includes all independent variables, with the exception of sex.

Threshold values of the test statistic were derived by permutation tests [19], using 1000 repetitions. All permutations were calculated for different traits in data sets for crosses and chromosomes separately. Applying a Bonferroni correction [20], the P < 0.01 and P < 0.05 genome-wide thresholds were calculated for chromosomes 7, 16 and × and then averaged across the chromosomes and crosses, since the thresholds between the crosses and traits showed only slight differences (Additional file 3).

Testing multifactorial influences on selected traits, the additive and dominant components of significant QTL positions detected across all the chromosomes were included stepwise by using a multiple position model which included the environmental variables. Components with a significant proportion of the explained variance remained in the final model (see results in Table 5).

**Table 5:** Combined analysis of significant QTL positions ^a^

					Single locus^c^				Combined loci^d^	
Trait^b^, Cross	SSC	Position (cM)	F ratio	P	VF_2 _(%)	r^2 ^(%)	Additive effect	F ratio	P	Additive effect
SEFW, W × M	1	71.0	17.3	< 0.001	4.8	18.0	-0.11	4.3	0.039	-0.05
	1	137.1	21.9	< 0.001	6.1	19.1	-0.12	16.4	< 0.001	-0.10
	5	73.0	18.2	< 0.001	5.0	18.2	0.10	15.0	< 0.001	0.08
	7	85.9	18.1	< 0.001	5.0	18.2	-0.10	18.8	< 0.001	-0.09
	X	90.0	15.3	< 0.001	4.2	17.5	-0.11	15.4	< 0.001	-0.11
			Initial model: r^2 ^(%) 13.6 Combined loci: VF_2 _(%) 20.2; r^2 ^(%) 32.1							
FD10, W × M	1	112.9	19.3	< 0.001	5.3	14.9	-2.18	14.9	< 0.001	-1.76
	2	46.5	13.7	< 0.001	3.8	13.5	-1.95	12.6	< 0.001	-1.70
	7	78.8	33.9	< 0.001	9.2	18.4	2.73	35.3	< 0.001	2.60
	X	90.0	20.5	< 0.001	5.7	15.2	-2.98	20.2	< 0.001	-2.71
			Initial model: r^2 ^(%) 9.8 Combined loci: VF_2 _(%) 19.4; r^2 ^(%) 30.2							
FMR, M × P	1	166.3	25.5	< 0.001	7.4	29.0	0.09	23.7	< 0.001	0.08
	2	0.0	14.7	< 0.001	4.3	26.6	0.06	9.5	0.002	0.04
	2	63.4	17.1	< 0.001	5.4	27.2	0.07	14.5	< 0.001	0.06
	6	96.9	35.6	< 0.001	10.1	31.1	0.10	32.3	< 0.001	0.09
			Initial model: r^2 ^(%) 23.1 Combined loci: VF_2 _(%) 22.8; r^2 ^(%) 41.4							
FV146, W × P	2	96.3	9.8	0.002	3.0	17.9	-2.96	11.3	< 0.001	-3.00
	4	76.9	19.3	< 0.001	6.0	20.4	4.35	17.7	< 0.001	4.00
	7	105.4	9.9	0.002	3.0	17.9	-3.71	8.9	0.003	-3.32
	X	0.0	9.5	0.002	2.9	17.8	3.06	11.9	< 0.001	3.23
			Initial model: r^2 ^(%) 15.0 Combined loci: VF_2 _(%) 14.4; r^2 ^(%) 28.3							
FVCM, M × P	1	207.3	8.7	0.003	2.7	16.0	-8.23	11.0	0.001	-8.48
	2	59.4	10.1	0.002	3.0	16.4	-5.34	13.6	< 0.001	-5.71
	7	74.8	37.1	< 0.001	10.8	23.2	10.31	42.3	< 0.001	10.56
	X	3.0	7.4	0.014	2.1	15.7	5.26	5.3	0.022	4.08
			Initial model: r^2 ^(%) 13.6 Combined loci: VF_2 _(%) 18.6; r^2 ^(%) 30.6							

Examples are given for some traits and show the results gained by including several genome-wide significant QTL across chromosomes

^a ^multiple position models were included together with the same environmental independent variables as in the initial model; ^b ^trait acronym, for definition see Table 2; ^c ^each QTL position was analyzed separately for trait association; *F *ratio: mean square estimates of the additive and dominant components in relation to the error variance of the model; VF_2_: proportion of error variance reduction by inclusion of additive and dominant components in the initial model; r^2^: determination coefficient; ^d ^QTL positions analyzed in combination

## Results and Discussion

### Genome-wide distribution of QTL

Within each cross, we identified QTL which explain more than about 4.3% of the error variance (VF_2_) with a P < 0.05 genome-wide significance level (threshold with *F *ratio > 8.5). As shown in Table 4, a total of 147 genome-wide QTL were found (76 at P < 0.05, and 71 at P < 0.01) for the three crosses. The numbers of significant QTL were 30 at P < 0.05 and 33 at P < 0.01 for M × P, 22 at P < 0.05 and 25 at P < 0.01 for W × P, and 24 at P < 0.05 and 13 at P < 0.01 for W × M. However, since we tested three populations and 40 traits in 120 genome scans, about six false positive QTL may occur at a genome-wide 5% significance.

The numbers of QTL detected per trait were about three times higher for the performance traits (Table 2a) than for the other groups of traits (protein, enzyme, fat cell traits, Table 2b and 2c). This finding can be explained by the fact that performance traits are likely to be influenced by a higher number of genes than protein, enzyme and fat cell traits.

**Figure 1:**
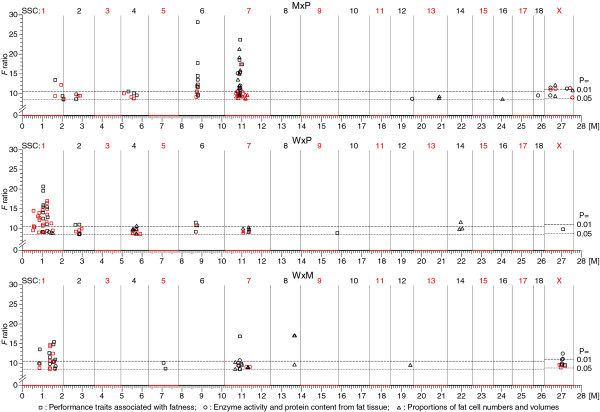
**Genomic distribution of QTL**. The distribution of the QTL detected in the Hohenheim crosses (M: Meishan; P: Pietrain; W: European wild boar) and with F ratio values above the genome-wide thresholds P = 0.05 is shown on the pig chromosomes (SSC); for each cross, the sex-averaged map in Kosambi morgan (M) is adjusted to the length calculated for the Hohenheim M × P cross; results for SSCX were obtained from female animals; the different symbols for the three trait groups represent major QTL peaks (black) and minor QTL peaks (red) that show distances > 20 cM to the major peak and to other minor peak observed for the same trait.

In Table 4, the QTL positions and the flanking marker loci for the Hohenheim maps are indicated together with the corresponding USDA MARC map positions. Figure 1 shows the genome-wide QTL distribution for the three crosses. For performance traits, if only the major QTL and adjusted positions on USDA MARC map are considered, the following results can be emphasized:

An accumulation of QTL for fat deposition traits (performance traits) was observed on **SSC1**. For the W × P cross, QTL were mainly located at positions 76-78 and 87-90 cM. QTL at positions 89-91 and 105-108 cM were detected in the W × M cross, besides two other QTL at positions 57 cM and 113 cM. QTL at 114 and 136 cM were observed in the M × P cross. A QTL for enzyme activity was found with a 5% significance level in the W × P cross, and several QTL were detected in W × P and W × M crosses for fat cell parameters at about 91, 104 and 111-113 cM, three of them near *SW705*, where [21] has detected QTL for fat cell traits.

On **SSC2**, only QTL related to performance traits were found in the W × P cross (at 57 cM and 73 cM) in spite of the fact that in the Pietrain breed, the allele *IGF2-intron3-3072A *responsible for a paternally expressed QTL at the proximal end (0.6 cM) of SSC2 affecting muscle growth and fat deposition is nearly fixed, while in wild boar and the Meishan breed only the wild allele *IGF2*-*intron3-3072G *is detected [22]. Therefore, F_1 _males from W × P and M × P crosses should be *IGF2 *heterozygous and about half of the F_2 _animals should possess the allele *IGF2-intron3-3072A*. The *IGF2-intron3-3072 *locus was not tested in the crosses as no suitable assay was available. However, its location corresponds to the interval between the markers *SW2443/SWC9 *and *S0141*, in which no QTL for performance traits was observed in this study.

Two QTL (P < 0.01) were detected on **SSC4**, one related to performance traits (37 cM, M × P cross) and one to fat cell traits (74 cM, W × P cross). Another QTL for fat cell traits was found at position 53-55 cM (W × P cross).

Several QTL for performance traits were also found on **SSC6 **in the M × P cross between the markers *TGFB1 *and *NPPB *at around 76 cM. The QTL for both traits on SSC6 in the W × P cross were located in the same interval. Whereas Bidanel et al. [23] have confirmed this QTL position, other authors [24,25] have mapped a QTL for back fat thickness on SSC6 in the vicinity of *SW1881 *corresponding to position 121 cM.

All 20 QTL (P < 0.01) on **SSC7 **were found in the major histocompatibility complex (*MHC*), of which 19 were located approximately 10 cM around the genes *TNFA *and *TNFB*. These 19 QTL seem to be distributed in three clusters, one slightly proximal to marker *KE6*, one slightly distal to *TNFA*/*TNFB *and one about 6 cM distal to *TNFA*/*TNFB*. The remaining QTL (performance trait AFW, M × P cross) was detected about 9 cM distal to *TNFA*/*TNFB*. A total of 18 QTL was observed in the M × P cross for performance (4), enzyme activity (5) and fat cell traits (9), and only two QTL were detected in the W × M cross (one for performance and one for enzyme activity traits). These differences of QTL between crosses might be affected by the information content of marker loci. The QTL for back fat thickness located near *TNFA/TNFB *have also been reported by [26-29] and Miller [21] has located QTL for fat cell traits at the same position.

On **SSC8, 12, 13, 14, 16 **and **18**, several QTL for traits related to protein content and fat cells were observed, three of them with P < 0.01. Amongst these, two concerning fat cell traits were found on **SSC8 **for the W × M cross at 108 cM (calculated from 91 observations only), and one QTL detected on **SSC14 **for another fat cell trait was located between the markers *SW210 *and *SW2488 *in the W × P cross.

QTL for protein content were detected on **SSCX **for the W × M cross at 80-81 cM in the immediate vicinity of *CAPN6*. QTL related to enzyme activities were found on SSCX in the M × P cross at positions 29, 57 and 112-114 cM. Another QTL for fat cell traits was found at about 56 cM, at the same position where [30] described a QTL for backfat thickness.

### Effects of F_2 _crosses on QTL profiles

As shown in Figure 1 and Table 4, most of the QTL were observed within a few chromosome regions only, and the QTL were often specific to one or two of the three F_2 _crosses. For example, QTL on SSCX occur mainly in the crosses M × P and W × M and with a cross-specific distribution. The QTL detected in similar chromosomal intervals in two of the three crosses indicate that alleles transmitted from one of the resource groups are different from the alleles in the two other resources.

High allelic effects caused by a distinct founder breed were observed, for example, on SSC4 (near *ATP1A2*), SSC6 (near *RYR1*) and SSC7 (between *TNFA *and *S0102*). The relevant SSC7 interval includes the MHC region where Meishan cryptic alleles are responsible for a decrease in fat deposition and enzyme activity traits and an increase in the proportion of small fat cells' numbers and volumes (observed in the F_2 _M × P and W × M crosses). The same effects of Meishan alleles on SSC7 have been reported for fat deposition as well as for numbers and volumes of adipocytes in a Large White × Meishan backcross [31]. On the contrary, Meishan alleles that increase fat deposition were located in the M × P and W × M crosses on SSC1 between *TGFBR1 *and *SW705*. Moreover, Pietrain alleles in the crosses with Meishan as well as with wild boar on SSC6 at *TGFB1/A1BG *had negative effects on obesity. None of the regions with significant effects on fat deposition traits was common to all three crosses, except the one for fat cell traits between *TNFB *and *PSMA4 *on SSC7 at about 55 to 90 cM referring to the USDA MARC map.

**Figure 2:**
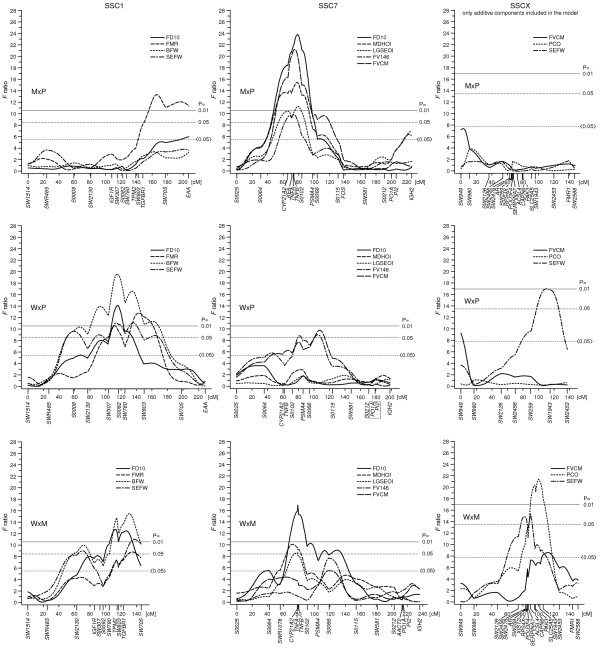
**Examples of F ratio profiles in the different Hohenheim crosses shown for chromosomes 1, 7 and X**. The solid line indicates the P = 0.01, the dashed line the P = 0.05 genome-wide thresholds, and the dotted line the P = 0.05 chromosome-wide threshold for F ratio values; traits are defined in Table 2; positions of markers are given in Kosambi centimorgan (cM) on the linkage maps of Hohenheim crosses; results for SSCX were obtained from female animals; markers are described in Additional file 1; data sets for the two sexes are shown with the averaged linkage map distances whereas for SSCX the female map distances are used; M: Meishan; P: Pietrain; W: European wild boar.

Figure 2 demonstrates the cross-specific QTL profiles for SSC1, SSC7 and SSCX. The QTL for protein content on SSCX at *CAPN6 *(mapped at 81 cM on USDA MARC map, [18,32]) was observed only in the W × M cross. Numerous QTL profiles on SSC1 and SSC7 were similar between the M × P and W × M crosses indicating that allele effects in Meishan were highly different to those in Pietrain and wild boar. However, SSC7 QTL were similar among all three crosses for an interval between about 50 and 100 cM (which contains the MHC, see Figure 2), revealing that major QTL effects are caused by alleles that segregate in all the crosses.

Several of the *F *ratio profiles reveal multiple peaks per chromosome (Figure 2, Table 4). This might be caused by pleiotropic effects of the involved genes. However, multiple peaks in the *F *ratio profile of a trait per chromosome may also result from linkage disequilibria among alleles of linked loci in F_2 _animals, whereby the linkage disequilibrium increases while the distances between the considered loci decrease. Significances of QTL peaks can be influenced by different information contents of the marker loci used in the flanking regions of a QTL. Thus, more markers and multipoint regression analyses may help to determine the contribution of single QTL peaks to the total genetic variance of the trait considered.

Examples of multiple and cross-specific QTL peaks per chromosome are also shown in Figure 2 for SSC7. In the M × P and W × M crosses, the major QTL profiles on SSC7 span from about 55 to 90 cM (including the genes *CYP21A2, KE6, TNFA, TNFB*), and in the W × P cross the major QTL were found at about 105 cM (between *S0066 *and *S0115*). The 30 cM interval covering the largest QTL on SSC7 contains the MHC known to include numerous functional genes in man and mouse. In this interval, genome-wide significant QTL were detected especially in both Meishan crosses. Concerning fat deposition traits, this could be due mainly to a smaller difference between the purebred estimates for wild boar and Pietrain compared to that between these two breeds and the Meishan breed [10]. For instance, the difference in average back fat depth (ABFD) between Pietrain and wild boar was 2.13 mm, whereas it was 7.77 mm between Pietrain and Meishan and 9.90 mm between wild boar and Meishan. A further example of effects of crosses on the patterns and positions of QTL was observed for SSC6 in the region of the loci *LIPE, TGFB1, A1BG, EAH *and *NPPB *(USDA MARC map 75 to 80 cM, Table 4). Important QTL were detected in this region for both M × P and W × P crosses. The additive effects for the grand-paternal inheritance indicate a negative influence of distinct Pietrain founder alleles on performance traits associated with fatness.

### Differences of QTL profiles calculated separately for female and male F_2 _offspring

**Figure 3:**
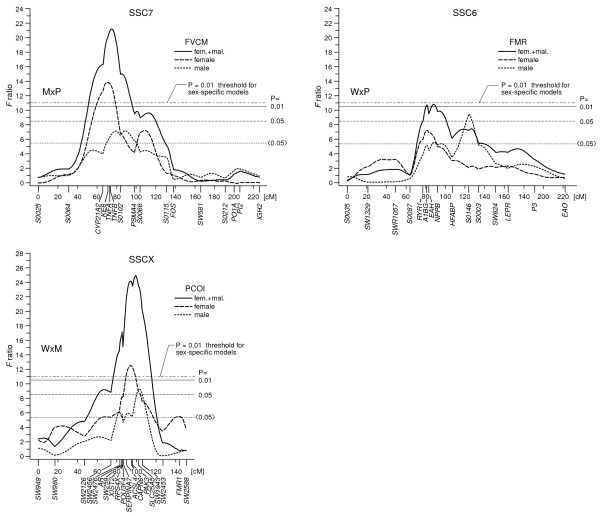
**Examples of F ratio profiles calculated for all (females and males), female or male F_2 _animals**. For further explanations see Figure 2.

QTL analyses for female and male F_2 _offspring are shown for example on SSC6, SSC7 and SSCX and use averaged linkage map distances for the autosomes and the female map distances for SSCX. Figure 3 shows QTL effects for the traits FVCM, FMR and PCOI, which differ between female and male F_2 _animals. For example, the trait FVCM in females of the M × P cross are highly influenced by a QTL at 75 cM on SSC7 whereas the *F *ratio value for males shows non-significance at that position. Males of the W × P cross show a QTL for the trait FMR on SSC6 at a position near 125 cM, which is located about 40 cM distal to the position found for all (female and male) animals. The trait PCOI represents an example of sex specific QTL positions on SSCX (QTL at 94 and 104 cM for females and males, respectively, W × M cross).

Sex specific QTL positions have also been reported on SSCX for muscle, fatness and growth related traits in the W × M cross [18]. Sex specific and fat related QTL have been described on chromosome 5 in chicken [33] and on several chromosomes in mouse [34]. Gene expression studies in male and female F_2 _mice have shown a large degree of sexually dimorphic gene expression in several tissues [35,36]. An expression QTL (eQTL) study [37] has shown that most of the eQTL were cis eQTL (mapping to the location of the gene) and sex-shared. Genetic mechanisms possibly underlying sex-specific expression, like sex linkage, sex-specific allelic effects or genomic imprinting, are discussed in [38].

### Combined analysis of significant QTL positions across chromosomes

Across all the pig chromosomes and for selected traits, we have carried out a combined analysis of the additive and dominant components of significant QTL positions. Taking each trait separately, the components of those positions were included step by step in a multiple position model. In the final model, only components with significant variance proportions were included. Examples of the results are shown in Table 5 and elucidate why the explained phenotypic variance in the F_2 _generations increased markedly up to about 23%, and the determination coefficients (r^2^) of the initial model (analyses without genetic independent variables) were more or less doubled. For each trait, several QTL positions, partially located on the same chromosome, remained significant in the combined analysis. This means that the combined analysis indicates a predominant contribution of a few QTL regions to the genetic variance of a trait. Therefore, multiple testing elucidates chromosome intervals which can be significant for breeding programmes.

## Conclusions

As demonstrated in this report, in pig, fat related traits correspond to numerous specific QTL positions across the genome. For some of the traits associated with specific enzymes, protein content and cell structure in fat tissue, it is the first time that they are included in a QTL analysis. We have found that QTL positions differ between F_2 _crosses, and differ partly for their additive and dominant effects. Some of these QTL show sex specific effects. Many of the QTL profiles possess multiple peaks especially in regions with a high marker density, and confidence intervals mostly exceed 10 cM [39]. Therefore, QTL intervals are rarely narrowed down to a sufficiently small number of candidate loci to be able to suggest one as the most probable causative gene.

Nevertheless, porcine chromosome regions, which contain QTL, can be aligned with loci of expressed genes, as well as with orthologous genes in man and mouse using data from PigQTLdb ([4]; http://www.animalgenome.org/QTLdb/pig.html). Today, QTL intervals can be compared with the pig genome sequence data (Sscrofa9, Wellcome Trust Sanger Institute 2009, http://www.sanger.ac.uk/Projects/S_scrofa/) to investigate the action of single genes and their variants. The selection of putative causative genes may consider groups of genes that are regulated in parallel and are members of the same metabolic pathway. Thus, the results of genome-wide QTL mapping are important for subsequent analyses of specific genes as well as for selecting DNA markers for breeding purposes.

## Competing interests

The authors declare that they have no competing interests.

## Authors' contributions

HG is responsible for most of the concept and design, for finding funding, and for drafting the tables and manuscript. SC, AS and SP have carried out the genotyping of marker loci and revised the manuscript. HB performed the statistical analysis, created the figures and helped to draft the manuscript.

All authors have read and approved the final manuscript.

## Acknowledgements

The investigation was supported by the German Research Foundation (DFG, grant nos. Mu616/6 and Ge291/20), the EC programmes BRIDGE and INCO-Copernicus (Contract no. ERBIC15CT960902), the Czech Science Foundation (Grant No. 523/07/0353 and 523/06/1302), and the Institutional Research Plan of the IAPG AS CR (AV0Z50450515). The Meishan pigs used in the experiments originated from a population provided by the Wageningen Agricultural University and Euribrid, BV Boxmeer, The Netherlands.

## Supplementary Material

Additional file 1 **Markers used for linkage and QTL analysis**. The used marker loci are shown together with literature references and positions on the USDA MARC map. Moreover, the map positions, numbers of alleles and numbers of informative meioses are listed for each of the three crosses. Click here for file

Additional file 2 **Parameters of the traits**. Numbers of observations, phenotypic means, standard deviations and determination coefficients are given for the considered traits of the F_2 _animals and each cross. Click here for file

Additional file 3 **Genome-wide threshold values**. The threshold values, which were calculated according to [19] and with 1000 permutations, are listed for the P < 0.05 and P < 0.01 significance levels. Click here for file
