# Supplementary material for: Genome-wide mapping of Quantitative Trait Loci for fatness, fat cell characteristics and fat metabolism in three porcine F2 crosses
Source: Genet Sel Evol. 2010 Jul 28;42(1):31. doi: 10.1186/1297-9686-42-31 (PMC2923101; doi:10.1186/1297-9686-42-31)
Supplement: Additional file 1 — Markers used for linkage and QTL analysis. The used marker loci are shown together with literature references and positions on the USDA MARC map. Moreover, the map positions, numbers of alleles and numbers of informative meioses are listed for each of the three crosses. [file 1297-9686-42-31-S1.DOCX]

**Suppl. Table 1** Markers used for linkage and QTL analysis

| SSC No. | Marker  Locus ^a^) | Type ^b^) | Reference | Average position (cM) ^c^) | | | | No. of alleles | | | No. of informative meioses ^d^) | | |
| --- | --- | --- | --- | --- | --- | --- | --- | --- | --- | --- | --- | --- | --- |
|  |  |  |  | MARC | MxP | WxP | WxM | MxP | WxP | WxM | MxP | WxP | WxM |
| **1** | *SW1514* | MS | Alexander et al., 1996a | 0.0 | 0 | 0 | 0 | 6 | 6 | 5 | 702 | 686 | 728 |
|  | *SWR485* | MS | Rohrer et al., 1994 | 16.4 | 24.1 | 27.5 | 25.2 | 6 | 5 | 5 | 595 | 625 | 590 |
|  | *S0008* | MS | Fredholm et al., 1993 | 43.5 | 58.8 | 58.7 | - | 6 | 5 | - | 244 | 715 | - |
|  | *SW2130* | MS | Alexander et al., 1996a | 49.4 | 80.7 | 76.9 | 63.1 | 6 | 7 | 3 | 702 | 543 | 602 |
|  | *IGF1R* | SNP | Kopecny et al., 2002 | *67.9* | 108.0 | - | 85.7 | 2 | - | 2 | 598 | - | 608 |
|  | *SW307* | MS | Rohrer et al., 1994 | 73.0 | 114.7 | 103.7 | 92.9 | 3 | 4 | 4 | 343 | 657 | 360 |
|  | *S0082* | MS | Ellegren et al., 1993 | *77.3* | 121.9 | 117.2 | 97.2 | 3 | 3 | 4 | 632 | 480 | 604 |
|  | *SW780* | MS | Rohrer et al., 1994 | 81.0 | 127.3 | 125.3 | 105.0 | 3 | 3 | 4 | 422 | 512 | 668 |
|  | *TPM2* | SNP | Kopecny et al., 2002 | *91.5* | 139.0 | - | 114.7 | 2 | - | 2 | 600 | - | 670 |
|  | *SW803* | MS | Rohrer et al., 1994 | 94.3 | 142.1 | 150.2 | 117.3 | 3 | 2 | 3 | 632 | 548 | 670 |
|  | *TGFBR1* | SNP | Kopecny et al., 2002 | *99.6* | 148.2 | - | 123.2 | 2 | - | 2 | 630 | - | 438 |
|  | *SW705* | MS | Rohrer et al., 1994 | 122.6 | 177.2 | 197.8 | 146.7 | 3 | 4 | 4 | 632 | 720 | 730 |
|  | *EAA* | BG | Hojny and Hala, 1965 | *142.7* | 207.2 | 229.2 | - | 2 | 2 | - | 622 | 298 | - |
|  |  |  |  |  |  |  |  |  |  |  |  |  |  |
|  |  |  |  | MARC | MxP | WxP | WxM | MxP | WxP | WxM | MxP | WxP | WxM |
| **2** | *SW2443* | MS | Alexander et al., 1996b | 0.0 | 0 | 0 | 0 | 3 | 3 | 4 | 702 | 716 | 319 |
|  | *SWC9* | MS | Rohrer et al., 1996 | 0.6 | 4.6 | 5.8 | 5.1 | 2 | 2 | 3 | 279 | 349 | 319 |
|  | *SW2623* | MS | Alexander et al., 1996b | 9.8 | 16.2 | 13.7 | 14.6 | 3 | 4 | 5 | 702 | 684 | 699 |
|  | *S0141* | MS | Wilke et al., 1994 | 31.2 | 43.3 | 35.6 | 40.5 | 4 | 3 | 4 | 632 | 630 | 670 |
|  | *SW240* | MS | Rohrer et al., 1994 | 42.0 | 54.3 | 50.6 | 54.0 | 6 | 5 | 4 | 632 | 280 | 670 |
|  | *MLP* | SNP | Jacobs et al. unpublished | *56.1* | 71.0 | - | - | 2 | - | - | 391 | - | - |
|  | *MYOD1* | SNP | Knoll et al., 1997 | *57.4* | 74.0 | 69.3 | 68.3 | 2 | 2 | 2 | 507 | 87 | 670 |
|  | *RETN (RSTN)* | SNP | Cepica et al., 2002 | *64.1* | 80.3 | - | 73.7 | 2 | - | 2 | 440 | - | 370 |
|  | *SW395* | MS | Rohrer et al., 1994 | 66.1 | 82.8 | 83.3 | 75.4 | 5 | 4 | 5 | 520 | 564 | 670 |
|  | *S0010* | MS | Fredholm et al., 1993 | 77.9 | 95.6 | 99.0 | 88.7 | 6 | 5 | 5 | 702 | 713 | 732 |
|  | *S0378* | MS | Robic et al., 1997 | *96.5* | 112.1 | 121.0 | 108.0 | 6 | 5 | 3 | 632 | 486 | 670 |
|  | *FBN2* | SNP | Stratil et al., 2008 | *100.5* | 116.5 | - | 112.1 | 2 | - | 2 | 428 | - | 534 |
|  | *SW2192* | MS | Alexander et al., 1996a | 116.2 | 132.5 | 143.1 | 126.9 | 4 | 2 | 3 | 603 | 99 | 438 |
|  | *S0036* | MS | Brown et al., 1994 | 132.1 | 153.1 | 173.5 | 147.3 | 3 | 4 | 3 | 258 | 687 | 732 |
|  |  |  |  |  |  |  |  |  |  |  |  |  |  |
|  |  |  |  | MARC | MxP | WxP | WxM | MxP | WxP | WxM | MxP | WxP | WxM |
| **3** | *SW72* | MS | Rohrer et al., 1994 | 17.8 | 0 | 0 | 0 | 4 | 4 | 4 | 632 | 718 | 630 |
|  | *S0206* | MS | Robic et al., 1995 | 42.3 | 24.9 | 32.4 | 17.1 | 5 | 5 | 2 | 612 | 547 | 620 |
|  | *ASPN* | SNP | Stratil et al., 2006 | *52.7* | 38.6 | - | - | 2 | - | - | 110 | - | - |
|  | *OGN (OIF)* | MS | Stratil et al., 2006 | *53.2* | 39.2 | - | - | 3 | - | - | 554 | - | - |
|  | *SW902* | MS | Rohrer et al., 1994 | 58.4 | 46.1 | 52.7 | 38.6 | 6 | 5 | 3 | 702 | 216 | 668 |
|  | *SW828* | MS | Alexander et al., 1996a | 64.8 | 62.8 | 70.7 | 53.6 | 5 | 3 | 4 | 632 | 593 | 732 |
|  | *SW314* | MS | Rohrer et al., 1994 | 90.6 | 92.9 | 99.5 | 86.4 | 5 | 6 | 4 | 503 | 572 | 666 |
|  | *APOB (LPB)* | AL | Rapacz et al., 1994 | 96.8 | 101.2 | 106.5 | 103.5 | 3 | 3 | 4 | 434 | 541 | 686 |
|  | *SW349* | MS | Rohrer et al., 1994 | 112.6 | 117.3 | 130.7 | 131.2 | 5 | 4 | 4 | 664 | 689 | 637 |
|  | *SW2532* | MS | Alexander et al., 1996a | 129.3 | 135.0 | 157.8 | 146.4 | 5 | 3 | 2 | 632 | 382 | 670 |
|  |  |  |  |  |  |  |  |  |  |  |  |  |  |
|  |  |  |  | MARC | MxP | WxP | WxM | MxP | WxP | WxM | MxP | WxP | WxM |
| **4** | *SW489* | MS | Rohrer et al., 1994 | 8.0 | 0 | 0 | 0 | 5 | 5 | 4 | 685 | 438 | 593 |
|  | *MYC ( CMYC)* | SNP | Reiner et al., 2000b | *21.7* | 21.7 | 17.9 | 19.3 | 2 | 2 | 2 | 610 | 503 | 427 |
|  | *SW835* | MS | Rohrer et al., 1994 | 27.1 | 25.9 | 31.6 | 24.5 | 3 | 3 | 3 | 632 | 686 | 515 |
|  | *SWR73* | MS | Alexander et al., 1996a | 40.5 | 42.2 | 39.0 | 41.4 | 3 | 2 | 2 | 632 | 40 | 630 |
|  | *SW2128* | MS | Alexander et al., 1996a | 48.0 | - | 55.1 | - | - | 2 | - | - | 416 | - |
|  | *S0145* | MS | Wilke et al., 1994 | 49.0 | 46.9 | - | 50.8 | 4 | - | 5 | 698 | - | 652 |
|  | *SW1073* | MS | Rohrer et al., 1994 | 62.3 | 57.0 | 63.9 | 64.3 | 5 | 3 | 4 | 700 | 284 | 663 |
|  | *SW1089* | MS | Rohrer et al., 1994 | 69.6 | 63.2 | 68.3 | 69.0 | 3 | 2 | 4 | 700 | 387 | 728 |
|  | *V-ATPase (VATP)* | SNP | Blazkova et al., 2000 | 72.0 | 65.9 | 69.8 | - | 2 | 2 | - | 101 | 197 | - |
|  | *ATP1B1* | SNP | Blazkova et al., 2000 | *73.4* | 68.9 | - | 72.3 | 2 | - | 2 | 630 | - | 341 |
|  | *S0073* | MS | Fredholm et al., 1993 | 74.4 | 74.3 | 76.8 | 73.6 | 4 | 5 | 4 | 432 | 337 | 533 |
|  | *SDHC* | SNP | Stratil et al., 2001b | *76.1* | 77.1 | - | - | 2 | - | - | 585 | - | - |
|  | *MPZ* | SNP | Wagenknecht et al., 2005 | *76.1* | 77.1 | - | - | 2 | - | - | 585 | - | - |
|  | *APOA2* | MS | Knoll et al., 2003 | *76.1* | 77.2 | - | - | 3 | - | - | 628 | - | - |
|  | *CASQ1* | SNP | Knoll et al., 2002 | *76.8* | 78.3 | - | - | 2 | - | - | 612 | - | - |
|  | *OCT1* | SNP | Sternstein and Brunsch unpublisched | *76.8* | - | 80.9 | - | - | 2 | - | - | 99 | - |
|  | *ATP1A2* | SSCP | Blazkova et al., 2000 | *77.6* | 79.4 | 83.8 | 76.8 | 2 | 2 | 2 | 382 | 275 | 266 |
|  | *MEF2D* | SNP | Wagenknecht et al., 2003 | *78.0* | 80.3 | - | - | 2 | - | - | 187 | - | - |
|  | *LMNA* | SNP | Wagenknecht et al., 2006 | *79.2* | 82.4 | - | - | 2 | - | - | 235 | - | - |
|  | *GBA* | SNP | Stratil et al., 2004 | *79.2* | 83.1 | - | 79.4 | 2 | - | 2 | 366 | - | 92 |
|  | *PKLR* | SNP | Knoll et al., 2000a | *80.2* | 83.6 | 88.9 | 80.0 | 2 | 2 | 2 | 379 | 466 | 662 |
|  | *IVL* | SNP | Blazkova et al., 2002 | *81.2* | 85.1 | - | 83.6 | 2 | - | 2 | 404 | - | 265 |
|  | *EAL* | BG | Hojny et al., 1966;  Linhart, 1971 | *84.6* | 93.2 | 92.7 | 87.4 | 4 | 4 | 3 | 696 | 64 | 656 |
|  | *ATP1A1* | SNP | Blazkova et al., 2002 | *86.5* | 95.1 | - | 89.9 | 2 | - | 2 | 612 | - | 613 |
|  | *AMPD1* | SNP | Stratil et al., 2000 | *88.1* | 97.7 | - | 92.3 | 2 | - | 2 | 632 | - | 568 |
|  | *NGFB* | DGGE | Kopecny et al., 2000 | 88.3 | 97.7 | 100.5 | 92.8 | 2 | 2 | 2 | 103 | 308 | 273 |
|  | *TSHB* | SNP | Knoll et al., 2000b | *91.3* | 100.1 | - | - | 2 | - | - | 257 | - | - |
|  | *SW2435* | MS | Alexander et al., 1996b | 102.8 | 109.2 | 106.6 | 100.7 | 3 | 2 | 5 | 630 | 336 | 728 |
|  | *AGL* | Indel | Stratil et al., 2003 | *113.4* | 121.4 | - | - | 2 | - | - | 539 | - | - |
|  | *S0097* | MS | Ellegren et al., 1993 | *126.5* | 133.8 | 131.7 | 130.2 | 4 | 3 | 5 | 664 | 595 | 726 |
|  | *ABCD3 (PXMP1)* | SNP | Stratil et al., 2001a | *136.5* | 137.3 | 135.0 | 156.6 | 2 | 2 | 2 | 311 | 424 | 257 |
|  |  |  |  |  |  |  |  |  |  |  |  |  |  |
|  |  |  |  | MARC | MxP | WxP | WxM | MxP | WxP | WxM | MxP | WxP | WxM |
| **5** | *SW413* | MS | Rohrer et al., 1994 | 8.4 | 0 | 0 | 0 | 6 | 4 | 3 | 700 | 654 | 483 |
|  | *SWR453* | MS | Rohrer et al., 1994 | 57.9 | 38.2 | 42.7 | 36.3 | 5 | 4 | 3 | 660 | 450 | 670 |
|  | *SW2425* | MS | Alexander et al., 1996b | 72.3 | 51.1 | 57.9 | 50.3 | 4 | 6 | 4 | 326 | 666 | 732 |
|  | *SW2* | MS | Rohrer et al., 1994 | 78.7 | 62.2 | 67.9 | 63.0 | 5 | 6 | 4 | 464 | 500 | 728 |
|  | *S0005* | MS | Fredholm et al., 1993 | 88.2 | 75.8 | 79.7 | 76.5 | 8 | 8 | 4 | 702 | 718 | 732 |
|  | *SW152* | MS | Rohrer et al., 1994 | 107.0 | 90.0 | 94.3 | 91.7 | 3 | 4 | 5 | 475 | 695 | 732 |
|  | *IGF1* | MS | Kirkpatrick, 1992 | 118.7 | 109.9 | - | 106.5 | 4 | ~~-~~ | 4 | 667 | ~~-~~ | 730 |
|  | *SW995* | MS | Rohrer et al., 1994 | 125.0 | 118.3 | 115.1 | 111.9 | 4 | 5 | 4 | 596 | 689 | 732 |
|  | *DCN* | SNP | Stratil et al., 2008 | *131.8* | 129.0 | - | - | 2 | - | - | 624 | - | - |
|  | *MYF5* | SNP | Stratil and Cepica, 1999 | *141.5* | 146.5 | 139.1 | 144.9 | 2 | 2 | 2 | 581 | 562 | 240 |
|  | *SW967* | MS | Rohrer et al., 1994 | 145.9 | 151.0 | 148.1 | 154.3 | 5 | 5 | 6 | 610 | 636 | 710 |
|  |  |  |  |  |  |  |  |  |  |  |  |  |  |
|  |  |  |  | MARC | MxP | WxP | WxM | MxP | WxP | WxM | MxP | WxP | WxM |
| **6** | *S0035* | MS | Brown and Archibald, 1995 | 7.3 | 0 | 0 | 0 | 6 | 6 | 4 | 702 | 720 | 732 |
|  | *SW1329* | MS | Alexander et al., 1996a | 18.6 | 24.6 | 21.4 | 29.3 | 4 | 3 | 5 | 671 | 705 | 658 |
|  | *SW1057* | MS | Rohrer et al., 1994 | 47.1 | 58.4 | 49.4 | 68.2 | 3 | 5 | 4 | 443 | 720 | 732 |
|  | *S0087* | MS | Ellegren et al., 1993 | 62.8 | 79.4 | 64.4 | 94.1 | 5 | 5 | 3 | 692 | 720 | 712 |
|  | *RYR1* | SNP | Otsu et al., 1992 | *72.4* | 95.0 | 77.2 | - | 2 | 2 | - | 622 | 630 | - |
|  | *ETH5001* | MS | Bolt et al., 1993 | *73.5* | - | - | 111.3 | - | - | 2 | - | - | 670 |
|  | *LIPE* | SNP | Knoll et al., 1998 | *75.6* | 96.9 | - | 115.4 | 2 | - | 2 | 387 | - | 389 |
|  | *TGFB1* | SNP | Kopecny et al., 2004 | *75.6* | 97.8 | - | - | 2 | - | - | 543 | - | - |
|  | *A1BG* | BP | Juneja et al., 1983 | *76.5* | 98.8 | 81.4 | 120.0 | 2 | 2 | 2 | 542 | 570 | 492 |
|  | *EAH* | BG | Hojny, 1973 | *77.3* | 100.4 | 83.3 | 120.0 | 3 | 3 | 3 | 407 | 562 | 507 |
|  | *SKI* | SNP | Stratil et al., 2002c | *79.9* | 103.9 | - | - | 2 | - | - | 296 | - | - |
|  | *NPPB (BNP1)* | SNP | Muladno et al., 1996 | *83.2* | - | 92.1 | 130.1 | - | 2 | 2 | - | 522 | 597 |
|  | *HFABP (FABP3)* | SNP | Gerbens et al., 1997 | *91.5* | 119.6 | 106.9 | 142.1 | 2 | 2 | 2 | 200 | 416 | 668 |
|  | *S0146* | MS | Wilke et al., 1994 | 102.2 | 135.9 | 124.1 | 157.3 | 3 | 3 | 2 | 362 | 628 | 386 |
|  | *S0003* | MS | Fredholm et al., 1993 | 102.0 | 145.0 | 134.0 | 163.8 | 5 | 6 | 2 | 589 | 720 | 554 |
|  | *SW824* | MS | Rohrer et al., 1994 | 110.4 | 161.6 | 151.6 | 176.6 | 4 | 4 | 3 | 632 | 490 | 732 |
|  | *LEPR-H* ^5^) | SNP | Stratil et al., 1998 | 122.0 | - | 163.7 | 188.6 | - | 2 | 2 | - | 403 | 252 |
|  | *LEPR-R* ^5^) | SNP | Stratil et al., 1998 | 122.0 | - | - | - | - | 2 | - | - | 161 | - |
|  | *P3* | AL | Matousek et al., 1986; Cepica et al., 1996 | *139.9* | 212.0 | 190.7 | - | 2 | 2 | - | 106 | 300 | - |
|  | *EAO* | BG | Hojny et al., 1966 | *156.9* | 232.1 | 222.3 | - | 2 | 2 | - | 249 | 524 | - |
|  |  |  |  |  |  |  |  |  |  |  |  |  |  |
|  |  |  |  | MARC | MxP | WxP | WxM | MxP | WxP | WxM | MxP | WxP | WxM |
| **7** | *S0025* | MS | Coppieters et al., 1993 | 3.7 | 0 | 0 | 0 | 4 | 2 | 5 | 698 | 241 | 732 |
|  | *S0064* | MS | Fredholm et al., 1993 | 30.2 | 31.1 | 36.2 | 41.6 | 3 | 4 | 5 | 626 | 693 | 633 |
|  | *SWR1078* | MS | Smith et al., 1995 | 32.9 | - | - | 55.9 | - | - | 4 | - | - | 732 |
|  | *CYP21A2-A* ^5^) | SNP | Knoll et al., 1998 | 58.9 | 66.3 | 60.4 | - | 2 | 2 | - | 115 | 286 | - |
|  | *CYP21A2-D* ^5^) | SNP | Knoll et al., 1998 | 58.9 | 66.3 | 60.4 | 78.3 | 2 | 2 | 2 | 276 | 294 | 662 |
|  | *KE6* | SNP | Jacobs et al. unpublisched | *56.3* | 71.0 | - | - | 2 | - | - | 317 | - | - |
|  | *TNF (TNFA)* | SNP | Knoll et al., unpublished | 57.7 | 73.2 | - | 78.8 | 2 | - | 2 | 216 | - | 377 |
|  | *TNFB* | MS | Rohrer et al., 1994 | 58.1 | 73.8 | 62.1 | 79.9 | 4 | 5 | 4 | 421 | 539 | 670 |
|  | *S0102* | MS | Ellegren et al., 1994 | 70.1 | 84.1 | 71.8 | 90.5 | 3 | 5 | 5 | 542 | 661 | 732 |
|  | *PSMA4* | SSCP | Davoli et al., 1998 | *77.9* | 98.4 | 86.1 | 105.2 | 4 | 3 | 3 | 626 | 527 | 664 |
|  | *S0066* | MS | Fredholm et al., 1993 | 82.8 | 104.3 | 94.4 | 118.5 | 2 | 3 | 2 | 618 | 328 | 670 |
|  | *S0115* | MS | Ruyter et al., 1994 | 102.2 | 131.3 | 123.8 | 153.8 | 7 | 5 | 4 | 702 | 608 | 732 |
|  | *FOS* | SNP | Reiner et al., 2000a | *106.5* | 138.3 | - | - | 2 | - | - | 606 | - | - |
|  | *SW581* | MS | Rohrer et al., 1994 | 123.8 | 166.4 | 148.0 | 181.0 | 3 | 2 | 3 | 617 | 332 | 668 |
|  | *S0212* | MS | Robic et al., 1994 | 141.2 | 190.9 | 170.7 | 202.8 | 4 | 5 | 3 | 620 | 720 | 647 |
|  | *AACT2 (AACT, (SERPINA3-2)* | SNP | Stratil et al., 2002b | *148.1* | - | - | 213.0 | - | - | 2 | - | - | 662 |
|  | *PO1A* | BP | Juneja and Gahne, 1987; Stratil et al., 1997a | *148.2* | 199.8 | 179.4 | 213.9 | 7 | 4 | 5 | 628 | 527 | 726 |
|  | *PI2* | BP | Juneja and Gahne, 1987; Stratil et al., 1997a | *149.9* | 206.1 | 179.4 | 214.5 | 4 | 3 | 3 | 628 | 455 | 165 |
|  | *IGH2* | AL | Rapacz and Hasler-Rapacz, 1982 | *164.7* | 226.2 | 198.9 | 235.9 | 2 | 2 | 2 | 262 | 450 | 664 |
|  |  |  |  |  |  |  |  |  |  |  |  |  |  |
|  |  |  |  | MARC | MxP | WxP | WxM | MxP | WxP | WxM | MxP | WxP | WxM |
| **8** | *SW905* | MS | Rohrer et al., 1994 | 20.8 | *0* | 0 | 0 | 3 | 5 | 6 | 630 | 718 | 728 |
|  | *PGCMUT* | SNP | Jacobs et al. unpublished | *34.5* | *17.3* | - | - | 2 | - | - | 278 | - | - |
|  | *SW933* | MS | Rohrer et al., 1994 | 46.3 | *32.3* | 29.1 | 34.1 | 4 | 3 | 3 | 632 | 532 | 670 |
|  | *SW1070* | MS | Rohrer et al., 1994 | 56.1 | *50.6* | 41.8 | 50.4 | 2 | 4 | 4 | 378 | 718 | 732 |
|  | *S0144* | MS | Jung et al., 1994 | 96.3 | *89.4* | 77.1 | 84.1 | 3 | 3 | 4 | 667 | 626 | 670 |
|  | *SW16* | MS | Rohrer et al., 1994 | *105.8* | *111.1* | 104.6 | 110.5 | 5 | 4 | 3 | 692 | 657 | 732 |
|  | *SW61* | MS | Rohrer et al., 1994 | 112.3 | *125.0* | 125.2 | 127.5 | 6 | 6 | 5 | 702 | 718 | 711 |
|  | *SPP1 (OPN)* | MS | Rohrer et al., 1994 | 120.2 | *153.1* | 154.7 | 145.4 | 4 | 4 | 5 | 484 | 718 | 732 |
|  |  |  |  |  |  |  |  |  |  |  |  |  |  |
|  |  |  |  | MARC | MxP | WxP | WxM | MxP | WxP | WxM | MxP | WxP | WxM |
| **9** | *EAK* | BG | Hojny et al., 1979; Nielsen and Vögeli, 1982 | *-7.2* | 0 | 0 | 0 | 4 | 4 | 4 | 614 | 456 | 660 |
|  | *HPX* | BP | Kalab and Stratil, 1989 | *7.8* | 19.0 | 18.9 | 20.8 | 3 | 4 | 5 | 645 | 501 | 664 |
|  | *SW21* | MS | Alexander et al., 1996a | 15.1 | 25.5 | 31.7 | 30.1 | 3 | 3 | 3 | 499 | 446 | 296 |
|  | *SW911* | MS | Rohrer et al., 1994 | 36.8 | 51.9 | 68.2 | 58.9 | 5 | 4 | 4 | 500 | 551 | 710 |
|  | *SLN* | SNP | Fontanesi et al., 2001 | *53.3* | 66.2 | - | 70.5 | 2 | - | 2 | 451 | - | 656 |
|  | *SW2074* | MS | Alexander et al., 1996a | 65.4 | 77.5 | 85.6 | 78.1 | 5 | 5 | 3 | 609 | 616 | 668 |
|  | *APOA1* | MS | Cymerowska-Prokopczyk et al., 1999 | 66.7 | 89.2 | 93.4 | 86.8 | 4 | 3 | 4 | 447 | 472 | 442 |
|  | *LPR* | AL | Rapacz et al., 1986 ;  Hojny et al., 1993 | *81.9* | 111.9 | 110.6 | 110.2 | 2 | 2 | 2 | 624 | 16 | 664 |
|  | *EAN* | BG | Saison, 1967 | *84.6* | 116.1 | 110.6 | - | 2 | 2 | - | 451 | 526 | - |
|  | *SW1435* | MS | Alexander et al., 1996a | 96.0 | 135.3 | - | 124.1 | 3 | - | 6 | 702 | - | 730 |
|  | *MYOG* | SNP | Cieslak et al., 2000 | *97.3* | 133.0 | 132.4 | 131.6 | 2 | 2 | 2 | 494 | 590 | 228 |
|  | *SW2093* | MS | Alexander et al., 1996a | *104.4* | 137.7 | 137.9 | ~~-~~ | 6 | 6 | ~~-~~ | 686 | 675 | ~~-~~ |
|  | *GLUL* | SNP | Stratil et al., 2002a | *110.5* | 147.9 | - | - | 2 | - | - | 519 | - | - |
|  | *SW174* | MS | Rohrer et al., 1994 | 122.9 | - | - | 159.6 | - | - | 2 | - | - | 335 |
|  | *S0114* | MS | Ruyter et al., 1994 | 122.9 | 158.7 | 162.4 | - | 5 | 4 | - | 486 | 467 | - |
|  | *EAE* | BG | Hojny and Nielsen, 1992 | *137.4* | 180.9 | 187.1 | 196.5 | 5 | 5 | 3 | 598 | 495 | 623 |
|  | *SW1349* | MS | Alexander et al., 1996a | 142.5 | 188.8 | 193.2 | 203.8 | 9 | 5 | 5 | 680 | 660 | 726 |
|  |  |  |  |  |  |  |  |  |  |  |  |  |  |
|  |  |  |  | MARC | MxP | WxP | WxM | MxP | WxP | WxM | MxP | WxP | WxM |
| **10** | *SW830* | *MS* | Rohrer et al., 1994 | 0.0 | 0 | 0 | 0 | 5 | 3 | 4 | 596 | 591 | 670 |
|  | *SW443* | *MS* | Rohrer et al., 1994 | 20.4 | 29.0 | 32.1 | 30.6 | 5 | 7 | 5 | 632 | 718 | 728 |
|  | *SW497* | *MS* | Rohrer et al., 1994 | 39.3 | 53.1 | 53.2 | 51.4 | 5 | 4 | 4 | 700 | 679 | 728 |
|  | *SWR1849* | *MS* | Alexander et al., 1996a | 65.1 | 80.2 | 80.1 | 78.2 | 5 | 4 | 5 | 700 | 576 | 732 |
|  | *SW2000* | *MS* | Alexander et al., 1996a | 86.3 | 105.6 | 105.4 | 97.7 | 4 | 4 | 3 | 630 | 622 | 670 |
|  | *SW1708* | *MS* | Alexander et al., 1996a | 101.0 | 128.3 | 125.5 | 113.3 | 7 | 8 | 6 | 698 | 570 | 730 |
|  | *SW2067* | *MS* | Alexander et al., 1996a | 128.0 | 153.1 | 156.2 | 136.4 | 6 | 6 | 6 | 630 | 699 | 730 |
|  |  |  |  |  |  |  |  |  |  |  |  |  |  |
|  |  |  |  | MARC | MxP | WxP | WxM | MxP | WxP | WxM | MxP | WxP | WxM |
| **11** | *S0392* | *MS* | Riquet et al., 1995 | 1.9 | 0 | 0 | - | 5 | 5 | - | 314 | 680 | - |
|  | *SW1632* | *MS* | Alexander et al., 1996a | 16.6 | 36.5 | 22.3 | 0 | 6 | 6 | 4 | 630 | 626 | 670 |
|  | *SW435* | *MS* | Rohrer et al., 1994 | 53.3 | 70.9 | 65.9 | 27.9 | 6 | 4 | 4 | 700 | 687 | 730 |
|  | *SW1827* | *MS* | Alexander et al., 1996a | 76.2 | 105.3 | 95.9 | 58.8 | 4 | 4 | 3 | 639 | 416 | 440 |
|  |  |  |  |  |  |  |  |  |  |  |  |  |  |
|  |  |  |  | MARC | MxP | WxP | WxM | MxP | WxP | WxM | MxP | WxP | WxM |
| **12** | *S0143* | MS | Wilke et al., 1994 | 6.6 | 0 | 0 | 0 | 4 | 3 | 3 | 464 | 582 | 646 |
|  | *EAD* | BG | Hradecky and Linhart, 1970 | *18.5* | 8.3 | - | 17.2 | 2 | - | 2 | 626 | - | 153 |
|  | *SW957* | MS | Rohrer et al., 1994 | 33.4 | - | - | 39.5 | - | - | 3 | - | - | 732 |
|  | *GH1-H* | SNP | Larsen and Nielsen, 1993 | 45.2 | 34.0 | 30.3 | 48.8 | 4 | 4 | 3 | 666 | 243 | 668 |
|  | *GH1-A* | SNP | Larsen and Nielsen, 1993 | *45.2* | 34.0 | 30.3 | - | 2 | 2 | - | 252 | 236 | - |
|  | *S0083* | MS | Ellegren et al., 1993 | *53.1* | - | 41.0 | - | - | 6 | - | - | 698 | - |
|  | *SW874* | MS | Rohrer et al., 1994 | 64.7 | 57.3 | 56.7 | 68.8 | 3 | 4 | 4 | 632 | 229 | 670 |
|  | *S0090* | MS | Ellegren et al., 1993 | 80.2 | 78.6 | 71.9 | 89.0 | 4 | 4 | 3 | 632 | 626 | 732 |
|  | *S0147* | MS | Wilke et al., 1994 | 89.9 | - | 85.0 | 107.9 | - | 4 | 2 | - | 662 | 328 |
|  | *S0106* | MS | Ellegren et al., 1994 | 95.8 | - | - | 121.5 | - | - | 4 | - | - | 730 |
|  | *SWR1021* | MS | Rohrer et al., 1994 | 113.1 | 109.3 | 104.0 | 143.7 | 5 | 6 | 3 | 440 | 640 | 523 |
|  | *SW605* | MS | Rohrer et al., 1994 | 108.3 | 116.8 | 111.1 | 158.8 | 5 | 4 | 3 | 702 | 143 | 670 |
|  |  |  |  |  |  |  |  |  |  |  |  |  |  |
|  |  |  |  | MARC | MxP | WxP | WxM | MxP | WxP | WxM | MxP | WxP | WxM |
| **13** | *S0282* | MS | Davies et al., 1994 | 0.0 | 0 | 0 | 0 | 5 | 4 | 3 | 632 | 676 | 732 |
|  | *S0076* | MS | Wintero et al., 1994a | 27.9 | 29.5 | 42.9 | 42.5 | 3 | 3 | 2 | 295 | 562 | 513 |
|  | *SW864* | MS | Rohrer et al., 1994 | 43.1 | 56.3 | 64.3 | 62.5 | 4 | 2 | 5 | 632 | 209 | 670 |
|  | *SWR1008* | MS | Rohrer et al., 1994 | 53.0 | - | - | 74.4 | - | - | 4 | - | - | 732 |
|  | *TF* | BP | Cizova et al., 1993 | *54.5* | - | 81.9 | 87.1 | - | 3 | 3 | - | 690 | 726 |
|  | *S0068* | MS | Archibald et al., 1992 | 62.2 | 76.9 | 92.2 | 105.1 | 8 | 7 | 4 | 702 | 691 | 710 |
|  | *PIT1 (POU1F1)* | SNP | Tuggle et al., 1993 | 70.1 | 89.3 | 107.0 | - | 2 | 2 | - | 498 | 169 | - |
|  | *SW520* | MS | Rohrer et al., 1994 | 74.4 | 102.7 | 112.5 | 134.2 | 4 | 5 | 5 | 556 | 517 | 732 |
|  | *SW38* | MS | Rohrer et al., 1994 | 101.6 | 133.7 | 143.3 | 168.1 | 4 | 4 | 3 | 632 | 197 | 670 |
|  | *S0215* | MS | Robic et al., 1994 | 121.2 | 152.0 | - | 203.3 | 3 | - | 5 | 702 | - | 670 |
|  | *CSTB* | SNP | Russo et al., 2002 | *138.0* | 169.0 | - | 235.5 | 2 | - | 2 | 442 | - | 630 |
|  |  |  |  |  |  |  |  |  |  |  |  |  |  |
|  |  |  |  | MARC | MxP | WxP | WxM | MxP | WxP | WxM | MxP | WxP | WxM |
| **14** | *SW857* | MS | Rohrer et al., 1994 | 7.4 | 0 | 0 | - | 6 | 5 | - | 421 | 590 | - |
|  | *SW2038* | MS | Rohrer et al., 1996 | 19.3 | 16.5 | 15.8 | 0 | 6 | 6 | 3 | 632 | 673 | 732 |
|  | *SW540* | MS | Rohrer et al., 1994 | 31.5 | 33.0 | 34.8 | 15.5 | 5 | 6 | 2 | 632 | 630 | 670 |
|  | *ACTN2* | SNP | Schmitz et al., 1993 | *38.5* | 45.2 | - | 25.0 | 3 | - | 3 | 536 | - | 660 |
|  | *ACTA1* | SNP | Schmitz et al., 1994 | *41.9* | - | - | 34.3 | - | - | 2 | - | - | 668 |
|  | *SW210* | MS | Rohrer et al., 1994 | 46.3 | 55.2 | 50.7 | 42.2 | 4 | 4 | 4 | 632 | 470 | 732 |
|  | *SW2488* | MS | Alexander et al., 1996a | 67.0 | 75.0 | 75.9 | 61.6 | 5 | 4 | 6 | 702 | 649 | 732 |
|  | *SW55* | MS | Alexander et al., 1996a | 79.0 | 93.8 | - | 77.5 | 3 | - | 4 | 556 | - | 670 |
|  | *SW2515* | MS | Alexander et al., 1996b | 108.7 | 124.1 | 111.8 | 106.1 | 3 | 2 | 2 | 630 | 182 | 670 |
|  |  |  |  |  |  |  |  |  |  |  |  |  |  |
|  |  |  |  | MARC | MxP | WxP | WxM | MxP | WxP | WxM | MxP | WxP | WxM |
| **15** | *KS169* | MS | Wang et al., 2000 | 3.0 | 0 | 0 | 0 | 6 | 4 | 3 | 702 | 569 | 652 |
|  | *S0148* | MS | Wilke et al., 1994 | 34.6 | 30.5 | 16.5 | 14.9 | 5 | 4 | 2 | 692 | 590 | 467 |
|  | *EAG* | BG | Andresen and Wroblewski, 1961 | *42.7* | 42.1 | 28.5 | 22.8 | 2 | 2 | 2 | 543 | 201 | 662 |
|  | *SW964* | MS | Rohrer et al., 1994 | 50.7 | 53.5 | 37.4 | 33.4 | 5 | 4 | 5 | 665 | 235 | 732 |
|  | *SW15* | MS | Rohrer et al., 1994 | 65.1 | 66.9 | 41.9 | 43.3 | 4 | 2 | 4 | 702 | 263 | 732 |
|  | *SW2053* | MS | Alexander et al., 1996a | 81.1 | 82.9 | 62.1 | 67.3 | 7 | 7 | 4 | 586 | 709 | 532 |
|  | *SW1983* | MS | Alexander et al., 1996a | 101.5 | 107.5 | 91.8 | 95.0 | 6 | 7 | 4 | 632 | 533 | 670 |
|  |  |  |  |  |  |  |  |  |  |  |  |  |  |
|  |  |  |  | MARC | MxP | WxP | WxM | MxP | WxP | WxM | MxP | WxP | WxM |
| **16** | *S0111* | MS | Rohrer et al., 1996 | 0.0 | 0 | 0 | 0 | 3 | 5 | 3 | 631 | 680 | 702 |
|  | *SW1035* | MS | Alexander et al., 1996a | 15.1 | 20.8 | 21.1 | 21.6 | 4 | 3 | 3 | 632 | 487 | 670 |
|  | *SW419* | MS | Rohrer et al., 1994 | 24.3 | 32.4 | 30.2 | 36.7 | 4 | 3 | 3 | 702 | 478 | 670 |
|  | *S0077* | MS | Wintero et al., 1994b | *32.9* | 41.0 | 42.7 | 47.9 | 4 | 5 | 4 | 632 | 722 | 709 |
|  | *S0026* | MS | Coppieters et al., 1993 | 46.9 | 56.9 | 59.7 | 67.6 | 5 | 3 | 4 | 702 | 568 | 693 |
|  | *SWR2480* | MS | Rohrer et al.,1996 | 55.7 | 65.3 | 67.2 | 75.5 | 5 | 4 | 3 | 702 | 722 | 720 |
|  | *SPARC* | SNP | Stratil et al., 2008 | *67.4* | 73.8 | - | - | 2 | - | - | 626 | - | - |
|  | *S0061* | MS | Fredholm et al., 1993 | 92.6 | 92.0 | 95.4 | 102.8 | 4 | 5 | 3 | 621 | 648 | 542 |
|  |  |  |  |  |  |  |  |  |  |  |  |  |  |
|  |  |  |  | MARC | MxP | WxP | WxM | MxP | WxP | WxM | MxP | WxP | WxM |
| **17** | *SW335* | MS | Rohrer et al., 1994 | 0.0 | 0 | 0 | 0 | 3 | 3 | 3 | 568 | 705 | 730 |
|  | *SW1891* | MS | Alexander et al., 1996a | 17.3 | 13.3 | 4.1 | 3.6 | 4 | 7 | 2 | 622 | 679 | 181 |
|  | *S0296* | MS | Hoyheim et al., 1995 | 32.0 | 29.3 | 10.3 | 9.6 | 6 | 5 | 6 | 632 | 706 | 732 |
|  | *SW1920* | MS | Alexander et al., 1996a | 56.4 | 57.3 | 30.7 | 38.5 | 6 | 5 | 3 | 700 | 643 | 395 |
|  | *GHRH* | SNP | Nielsen and Larsen, 1997 | *58.7* | 58.5 | 34.1 | 43.0 | 2 | 2 | 2 | 489 | 135 | 290 |
|  | *SJ063* | MS | Wada et al., 2000 | *78.6* | 79.8 | 63.7 | 71.7 | 4 | 3 | 3 | 628 | 295 | 644 |
|  | *GNAS* | SNP | Stratil et al., 2008 | *88.5* | 95.6 | - | - | 2 | - | - | 184 | - | - |
|  | *SW2427* | MS | Alexander et al., 1996b | 97.0 | 105.8 | 86.9 | 95.7 | 4 | 7 | 4 | 581 | 722 | 732 |
|  |  |  |  |  |  |  |  |  |  |  |  |  |  |
|  |  |  |  | MARC | MxP | WxP | WxM | MxP | WxP | WxM | MxP | WxP | WxM |
| **18** | *SW1808* | MS | Alexander et al., 1996a | 0.0 | 0 | 0 | 0 | 5 | 7 | 4 | 630 | 718 | 732 |
|  | *EAI* | BG | Andresen, 1964 | *7.8* | 9.6 | - | 12.4 | 2 | - | 2 | 558 | - | 662 |
|  | *LEP (LEPTIN)* | SNP | Stratil et al., 1997b | *24.4* | 31.5 | - | 36.9 | 2 | - | 2 | 590 | - | 662 |
|  | *SW787* | MS | Rohrer et al., 1994 | 31.6 | 42.5 | 31.4 | 46.2 | 3 | 4 | 4 | 630 | 322 | 551 |
|  | *S0062* | MS | Fredholm et al., 1993 | 43.5 | 56.4 | 48.7 | 62.3 | 6 | 5 | 4 | 700 | 535 | 730 |
|  |  |  |  |  |  |  |  |  |  |  |  |  |  |
|  |  |  |  | MARC^e^ | MxP | WxP | WxM | MxP | WxP | WxM | MxP | WxP | WxM |
| **X** | *SW949* | MS | Rohrer et al., 1994 | 0.0 | 0 | 0 | 0 | 5 | 6 | 5 | 650 | 716 | 730 |
|  | *SW980* | MS | Rohrer et al., 1994 | 11.9 | 11.1 | 17.5 | 16.7 | 4 | 5 | 3 | 608 | 580 | 590 |
|  | *SW2126* | MS | Alexander et al., 1996a | 35.2 | 36.5 | 52.3 | 47.3 | 4 | 4 | 3 | 698 | 588 | 730 |
|  | *SW2456* | MS | Alexander et al., 1996b | 55.4 | 53.7 | 69.8 | 74.2 | 2 | 2 | 4 | 649 | 495 | 730 |
|  | *SW2476* | MS | Rohrer et al., 1996 | 77.6 | 62.6 | - | 82.6 | 4 | - | 3 | 542 | - | 730 |
|  | *AR* | MS | Rohrer, 1999 | *65.3* | 65.5 | - | 85.3 | 3 | - | 2 | 632 | - | 730 |
|  | *SW259* | MS | Rohrer et al., 1994 | *67.0* | 65.9 | 90.5 | 85.6 | 4 | 3 | 3 | 698 | 542 | 730 |
|  | *XIST* | SNP | Cepica et al., 2006 | 74.4 | 66.8 | - | 86.2 | - | - | 3 | - | - | 730 |
|  | *RPS4X* | SNP | Cepica et al., 2006 | 74.4 | 66.8 | - | 86.2 | 2 | - | 2 | 700 | - | 730 |
|  | *POU3F4* | SNP | Cepica et al., 2006 | 74.4 | 66.8 | - | 86.2 | 2 | - | 2 | 702 | - | 730 |
|  | *SERPINA7* | SNP | Cepica et al., 2001 | 75.5 | 68.1 | - | 86.5 | 2 | - | 2 | 700 | - | 730 |
|  | *ACSL4 (FACL4)* | SNP | Cepica et al., 2006 | *70.4* | 72.7 | - | 89.9 | 2 | - | 2 | 694 | - | 496 |
|  | *CAPN6* | SNP | Cepica et al., 2006 | *75.8* | 79.4 | - | 95.5 | 2 | - | 2 | 603 | - | 730 |
|  | *PAK3* | SNP | Cepica et al., 2006 | *76.5* | 80.1 | - | 96.4 | 2 | - | 2 | 519 | - | 722 |
|  | *SLC25A5 (ANT2)* | SNP | Cepica et al., 2001 | *83.9* | 91.3 | - | 102.4 | 2 | - | 2 | 698 | - | 730 |
|  | *SW1943* | MS | Alexander et al., 1996a | 87.4 | 95.7 | 115.2 | 106.0 | 3 | 3 | 2 | 643 | 701 | 730 |
|  | *SW2453* | MS | Alexander et al., 1996b | 107.8 | 120.3 | 137.4 | 126.9 | 2 | 3 | 2 | 418 | 716 | 730 |
|  | *FMR1* | SNP | Cepica et al., 2001 | *122.2* | 139.3 | - | 144.0 | 2 | - | 2 | 596 | - | 724 |
|  | *SW2588* | MS | Lopez-Corrales et al., 1999 | 128.4 | 147.4 | - | 151.6 | 3 | - | 3 | 587 | - | 730 |

F_2_ crosses: MxP, Meishan ♂ x Pietrain ♀; WxP, European Wild Boar ♂ x Pietrain ♀; WxM, European Wild Boar ♂ x Meishan ♀.

^a^) *IGFR (IGF1R)*: Insulin-like growth factor 1 receptor, *Sac*II-RFLP; *TPM2*: Tropomyosin 2, *Alu*I-RFLP; *TGFBR1*: Transforming growth factor-beta receptor, type I, *Hin*fI-RFLP; *EAA*: Erythrocyte Antigen A;*MLP*: LIM protein MLP gene, *Taq*I-RFLP; *MYOD1*: myogenin, myogenic factor 3, *Dde*I-RFLP; *RETN (RSTN)*: Resistin, *Mva*I-RFLP; *FBN2*: Fibrillin 2, *Hae*III-RFLP; *ASPN*: Asporin, *Taq*I-RFLP; *OGN (OIF)*: Osteoglycin, MS *SCZ003*; *APOB (LPB*): Apolipoprotein B (lipoprotein B); *MYC*: c-myc protooncogene protein, *Hpa*II-RFLP; *V-ATPase*: Vacuolar H(+) ATPase subunit gene, *Dra*I-RFLP; *ATP1B1*: Na^+^-K^+^-ATPase, β-subunit, *Hpa*II-RFLP; *SDHC* : Succinate dehydrogenase complex, subunit C, *Hin*fI-RFLP; *MPZ*: Myelin protein zero, *Rsa*I-RFLP; *APOA2*: Apolipoprotein A-II, MS *SCZ001*; *CASQ1*: Calsequestrin 1, *Alw*26I-RFLP; *OCT1*: organic cation transporter 1; *ATP1A2*: Na^+^-K^+^-ATPase, subunit α_2_; *MEF2D*: MADS box transcription enhancer factor 2, polypeptide D, *Aci*I-RFLP; *LMNA*: Lamin A/C, *Alu*I-RFLP; *GBA*: Glucocerebrosidase, *Hae*III-RFLP; *PKLR*: pyruvate kinase (liver and red blood cell), *Mva*I-RFLP; *IVL*: Involucrin, *Ava*II-RFLP (isoschizomer is *Eco*47I); *EAL*: erythrocyte antigen L; *ATP1A1*: ATPase, Na+/K+ transporting, alpha-1 polypeptide, *Hpa*II-RFLP; *AMPD1*: adenosine monophosphate deaminase 1, *Rsa*I-RFLP; *NGFB*: nerve growth factor β; *TSHB*: thyroid stimulating hormone, β-subunit, *Mn*lI-RFLP; *AGL*: Glycogen debranching enzyme, Indel; *ABCD3 (PXMP1)*: ATP-binding cassette, subfamily D, member 3, *Hpa*II-RFLP; *IGF1*: insulin-like growth factor I; *DCN*: Decorin, *Hpa*II-RFLP; *MYF5*: myogenic factor 5, *Hpa*II-RFLP; *RYR1*: ryanodine receptor 1 gene, *Hin*PI-RFLP; *LIPE*: hormone-sensitive lipase, *Hsp*92I-RFLP; *TGFB1*: Transforming growth factor, beta-1, *Ava*II-RFLP; *A1BG*: alpha 1B-glycoprotein (alias *PO2*); *EAH*: erythrocyte antigen H; *SKI*: v-ski avian sarcoma virol oncogene homolog; *Alw*26I-RFLP; *NPPB (BNP1)*: brain natriuretic peptide 1; *HFABP (FABP3)*: fatty acid-binding protein, heart; *LEPR-H*: leptin receptor, *Hpa*II-RFLP; *LEPR-R*: leptin receptor, *Rsa*I-RFLP; *P3*: uncharacterised allotype P3 (protein P3); *EAO*: erythrocyte antigen O; *CYP21A2-A*: Cytochrome P450, steroid 21-hydroxylase gene, *Alu*I-RFLP; *CYP21A2-D*: Cytochrome P450, steroid 21-hydroxylase gene, *Dde*I-RFLP; *KE6*: kappa light chain enhancer 6, *Bbv*I-RFLP; *TNF (TNFA)*: Tumor necrosis factor (TNF superfamily, member 2), *Nla*IV-RFLP; *TNFB*: Tumor Necrosis Factor β; *PSMA4*: proteasome subunit A4; *FOS*: c-Fos proto-oncogene; *SERPINA3-2 (AACT)*: alpha-1-antichymotrypsin 2; *PO1A*: Postalbumin 1A; *PI2*: α-Protease Inhibitor; *IGH2*: Immunoglobulin Gamma Heavy chain 2; *PGCMUT (PPARGC1)*: Peroxisome proliferative activated receptor gamma coactivator 1, *Ase*I-RFLP; *SPP1(OPN)*: Secreted phosphoprotein 1 (Osteopontin gene); *EAK*: erythrocyte antigen K; *HPX*: haemopexin; *SLN*: Sarcolipin; *APOA1*: apolipoprotein AI; *LPR*: lipoprotein R; *EAN*: erythrocyte antigen N; *MYOG*: myogenin, *Msp*I-RFLP; *GLUL*: Glutamate-ammonia ligase, *Hae*III-RFLP; *EAE*: erythrocyte antigen E; *EAD*: Erythrocyte Antigen D; *GH1-H*: Growth Hormone, *Hin*PI-RFLP; *GH1-A*: Growth hormone, *Apa*I-RFLP; *TF*: Transferrin; *PIT1 (POU1F1)*: POU domain class 1 transcription factor 1; *CSTB*: Cystatin B, *Alu*I-RFLP; *ACTN2*: Actinin, alpha 2; *ACTA1*: Actin, alpha 1; *EAG:* Erythrocyte Antigen G; *SPARC*: Secreted protein acidic and rich in cysteine, *Tas*I-RFLP; *GHRH*: Growth Hormone Releasing Hormone; *GNAS*: Guanine-nucleotide binding protein, alpha stimulating activity polypeptide 1, *Tai*I-RFLP; *EAI*: Erythrocyte Antigen I; *LEP*: Leptin; *AR*: Androgen Receptor; *XIST*: X (inactive)-specific transcript, *Alw*441-RFLP; *RPS4X*: Ribosomal protein S4, X-linked, *Pst*I-RFLP; *POU3F4*: POU class 3 homeobox 4, *Alu*I-RFLP; *SERPINA7 (TBG)*: serine (or cysteine) proteinase inhibitor a, member 7 (thyroxine-binding globulin), *Hin*fI-RFLP; *ACSL4 (FACL4)*: Acyl-CoA synthetase long-chain family member 4, *Taq*I-RFLP; *CAPN6*: Calpain 6, *Msp*I-RFLP; *PAK3*: P21 (CDKN1A)-activated kinase 3, *Hae*III-RFLP; *SLC25A5 (ANT2)*: adenine nucleotide translocase, *Alu*I-RFLP; *FMR1*: fragile mental retardation gene 1, *Fok*I-RFLP.

^b^) MS: Microsatellite; BG: Blood Group; AL: Allotype; SNP: Single Nucleotide Polymorphism; BP: Biochemical Polymorphism; SSCP: Single Strand Conformation Polymorphism; DGGE: Denaturant Gradient Gel Electrophoresis; Indel: Insertion or Deletion polymorphism.

^c^) Distances given in Kosambi cM for the sex averaged map.

MARC: USDA MARC map (reference map, http://www.marc.usda.gov/genome/swine/swin.html), positions in type regular represent original values in cM (Kosambi), italic letters represent interpolated values from flanking positions of the USDA MARC and the Hohenheim maps.

^d^) Informative meioses in the F_1_ and F_2_ generations.

**References**

Alexander, L.J., Rohrer, G.A., Beattie, C.W., 1996a: Cloning and characterization of 414 polymorphic porcine microsatellites. Anim. Genet. 27, 137-148.

Alexander, L.J., Troyer, D.L., Rohrer, G.A., Smith, T.P.L., Schook, L.B., Beattie, C.W., 1996b: Physical assignments of 68 porcine cosmid and lambda clones containing polymorphic microsatellites. Mamm. Genome 7, 368-372.

Andresen, E., Wroblewski, A., 1961: The G and H blood group systems of the pig. Acta Vet. Scand. 2, 267-280.

Andresen, E., 1964: The inheritance of the blood factors Ia and Ib in pig of the Duroc and Hampshire breeds. Vox Sang. 9, 617-621.

Archibald, A.L., Brown, J.F., Haley, C.S., Fredholm, M., Winterø, A.K., Coppieters, W., Van de Weghe, A., Signer, E., Larsen, N.J., Nielsen, V.H., Johansson, M., Andersson, L., 1992: Linkage mapping in the domestic pig (Sus scrofa). Anim. Genet. 23, Suppl. 1, p88.

Blazkova, P., Kopecny, M., Fontanesi, L., Stratil, A., Davoli, R., Reiner, G., Geldermann, H., 2000: Linkage assignments of the porcine ATP1A2, ATP1B1 and V-ATPase (CGI-11) genes to chromosome 4. Anim. Genet. 31, 416-418

Blazková P., Stratil A., Peelman L.J., Van Poucke M., Reiner G., Geldermann H., Kopecný M., 2002: Linkage assignments of the porcine ATP1A1 and IVL genes, and RH mapping of ATP1A1, ATP1B1, V-ATPase (CGI-11) and IVL to chromosome 4. Anim. Genet. 33, 235-237.

Bolt, R., Vögeli, P., Fries, R., 1993: A polymorphic microsatellite at the RYR1 locus in swine. Anim. Genet. 24, 72.

Brown, J.F., Hardge, T., Rettenberger, G., Archibald, A.L., 1994: Four new porcine polymorphic microsatellite loci (S0032, S0034, S0036, S0037). Anim. Genet. 25, 365.

Brown, J.E., Archibald, A.L., 1995: Two porcine polymorphic microsatellite loci (S0033 and S0035). Anim. Genet. 26, 277.

Čepica, S., Moser, G., Schröffel, J., Knorr, C., Geldermann, H., Stratil, A., Hojny, J., 1996: Chromosomal assignment of porcine EAD, EAO, LPR and P3 genes by linkage analysis. Anim. Genet. 27, 109-111.

Čepica, S., Rohrer, G.A., Knoll, A., Masopust, M., Malek, O., 2001: Linkage mapping of four genes (OTC, SERPINA7, SLC25A4 and FMR1) on porcine chromosome X. Anim. Genet., 32, 106-109.

Čepica, S., Rohrer, G.A., Masopust, M., Kubickova, S., Musilova, P., Rubes, J., 2002: Partial cloning, cytogenetic and linkage mapping of the porcine resistin (*RSTN*) gene. Anim. Genet. 33, 381-383.

Čepica, S., Masopust, M., Knoll, A., Bartenschlager, H., Yerle, M., Rohrer, G.A., Geldermann, H. (2006) Linkage and RH mapping of 10 genes to a QTL region for fatness and muscling traits on pig chromosome X. Anim. Genet., 37, 603-4.

Cieslak, D., Kapelanski, W., Blicharski, T., Pierzchala, M., 2000: Restriction fragment length polymorphisms in myogenin and myf3 genes and their influence on lean meat content in pigs. J. Anim. Breed. Genet. 117, 43-55.

Cizova, D., Stratil, A., Müller, E., Čepica, S., 1993: A new, partially deficient transferrin variant in the pig. Anim. Genet. 24, 305-306.

Coppieters, W., Van de Weghe, A., Peelman, L., Depicker, A., Van Zeveren, A., Bouquet, Y., 1993: Characterization of porcine polymorphic microsatellite loci. Anim. Genet. 24, 163-170.

Cymerowska-Prokopczyk, I., Pierzchala, M., Korwin-Kossakowska, A., 1999: The Polish “Pig Genome Mapping” project. VIII. Polymorphism of microsatellite sequences in the introns of apolipoprotein APOA1 and APOB genes. Animal Sci. Papers Reports 17, 35-44.

Davies, W., Høyheim, B., Chaput, B., Archibald, A.L., Frelat, G., 1994: Characterization of microsatellites from flow-sorted porcine chromosome 13. Mamm. Genome 5, 707-711.

Davoli, R., Fontanesi, L., Russo, V., Čepica, S., Musilova, P., Stratil, A., Rubes, J., 1998: The porcine proteasome subunit A4 (PSMA4) gene: isolation of a partial cDNA, linkage and physical mapping. Anim. Genet. 29, 385-388.

Ellegren, H., Johansson, M., Chowdhary, B.P., Marklund, S., Ruyter, D., Marklund, L., Bräuner-Nielsen, P., Edfors-Lilja, I., Gustavsson, I., Juneja, R.K., Andersson, L., 1993: Assignment of 20 microsatellite markers to the porcine linkage map. Genomics 16, 431-439.

Ellegren, H., Chowdhary, B., Johansson, M., Andersson, L., 1994: Integrating the porcine physical and linkage map using cosmid-derived markers. Anim. Genet. 25, 155-164.

Fontanesi, L., Davoli, R., Milc, J., Russo, V., 2001: The porcine sarcolipin (SLN) gene: identification of a SNP and linkage mapping to chromosome 9. Anim. Genet. 32, 109-110.

Fredholm, M., Winterø, A.K., Christensen, K., Kristensen, B., Nielsen, P.B., Davies, W., Archibald, A.L., 1993: Characterization of 24 porcine (dA-dC)n-(dT-dG)n microsatellites: Genotyping of unrelated animals from four breeds and linkage studies. Mamm. Genome 4, 187-192.

Gerbens, F., Rettenberger, G., Lenstra, J.A., Veerkamp, J.H., Te Pas, M.F., 1997: Characterization, chromosomal localization and genetic variation of the porcine heart fatty acid-binding protein gene. Mamm. Genome 8, 328-332.

Hojny, J., Hala, K., 1965: A contribution to the study of the blood group system A in pigs. In: Proc. 9th Europ. Anim. Blood Grps Conf. (Ed. J. Matousek), Prague, pp. 155-161.

Hojny, J., Gavalier, M., Hradecky, J., Linhart, J., 1966: New blood group factors in pigs. In: Polymorphismes biochimiques des animaux. INRA, Paris, p. 151-158.

Hojny, J., 1973: Further contribution to the H blood group system in pigs. Anim. Blood Grps. Biochem. Genet. 4, 161-168.

Hojný, J., Hradecky, J., Pazdera, J., 1979: The blood group factor Kf and allele K^ae^ in the pig. Anim. Blood Grps. Biochem. Genet. 10, 175-180.

Hojný, J., Nielsen, P.B., 1992: Allele E^bdgjmr^ (E^17^) in the pig E blood group system. Anim. Genet. 23, 523-524.

Hojný, J., Janik, A., Schröffel, J. Jr., 1993: A new lipoprotein allotype Lpr3 and allele Lpr1,3 in the pig. Anim. Genet. 24, 445-446.

Hoyheim, B., Keiserud, A., Thomsen, P.D., 1995: A highly polymorphic porcine dinucleotide repeat S0296 (BHT137) at chromosome 17q13. Anim. Genet. 26, 58.

Hradecky, J., Linhart, J., 1970: Db – next blood group factor of the D system in pig. Anim. Blood Grps Biochem. Genet. 1, 65-66.

Juneja, R.K., Gahne, B., Edfors-Lilja, I., Andresen, E., 1983: Genetic variation at a pig serum protein locus, Po-2 and its assignment to the Phi, Hal, S, H, Pgd linkage group. Anim. Blood Grps. Biochem. Genet. 14, 27-36.

Juneja, R.K., Gahne, B., 1987: Simultaneous phenotyping of pig plasma α-protease inhibitors (PI1, PO1A, PO1B, PI2) and four other proteins (PO2, TF, CP, HPX) by a simple method of 2D horizontal electrophoresis. Anim. Genet. 18, 197-211.

Jung, M., Chen, Y., Geldermann, H., 1994: Nine porcine polymorphic microsatellites (S0141 – S0149). Anim. Genet. 25, 378.

Kalab, P., Stratil, A., 1989: Phenotyping of pig α_1_B-glycoprotein (PO2) and haemopexin by 1D polyacrylamide gel electrophoresis and immunoblotting. Anim. Genet. 20, 295-298.

Kirkpatrick, B.W., 1992: Identification of a conserved microsatellite site in the porcine and bovine insulin-like growth factor-I gene 5´ flank. Anim. Genet. 23, 543-548.

Knoll, A., Nebola, M., Dvořák, J., Čepica, S., 1997: Detection of a DdeI PCR RFLP within intron 1 of the porcine MYOD1 (MYF3) locus. Anim. Genet. 28, 321.

Knoll, A., Stratil, A., Nebola, M., Čepica, S., 1998: Characterization of a polymorphism in exon 1 of the porcine hormone-sensitive lipase (LIPE) gene. Anim. Genet. 29, 462-463.

Knoll, A., Čepica, S., Stratil, A., Nebola, M., Dvorak, J., 1998: Numerous PCR-RFLPs within the porcine CYP21 (steroid 21-hydroxylase) gene. Anim. Genet. 29, 402-403.

Knoll, A., Stratil, A., Moser, G., Geldermann, H., 2000a: Characterization of three PCR-RFLPs in an intron of the porcine liver- and red cell-type pyruvate kinase (PKLR) gene. Anim. Genet. 31, 234.

Knoll, A., Stratil, A., Schröffel, J., Moser, G., Dvorak, J., Čepica, S., 2000b: A rare PCR-RFLP within intron 2 of the porcine TSHB gene. Czech. J. Anim. Sci. 45, 237-239.

Knoll A., Stratil A., Reiner G., Peelman L.J., Van Poucke M., Geldermann H., 2002: Linkage and radiation hybrid mapping of the porcine calsequestrin 1 (CASQ1 ) gene to chromosome 4q. Anim. Genet. 33, 390-2.

Knoll A., Stratil A., Vykoukalová Z., Van Poucke M., Bartenschlager H., Peelman L.J., Geldermann H., 2003: Polymorphism analysis and mapping to SSC4 of the porcine apolipoprotein A2 (APOA2) gene. Anim. Genet. 34, 384-6.

Kopecny, M., Stratil, A., Čepica, S., Moser, G., 2000: Polymorphism in the porcine NGFB gene detected by DGGE and its linkage mapping. Czech J. Anim. Sci. 45, 189-192.

Kopecný M., Stratil A., Bartenschlager H., Peelman L.J., Van Poucke M., Geldermann H., 2002: Linkage and radiation hybrid mapping of the porcine IGF1R and TPM2 genes to chromosome 1. Anim. Genet. 33, 398-400.

Kopecný M., Stratil A., Van Poucke M., Bartenschlager H., Geldermann H., Peelman L.J., 2004: PCR-RFLPs, linkage and RH mapping of the porcine TGFB1 and TGFBR1 genes. Anim. Genet. 35, 253-5.

Larsen, N.J., Nielsen, V.H., 1993: ApaI and CfoI polymorphisms in the porcine growth hormone gene. Anim. Genet. 24, 71.

Linhart, J., 1971: Lm, a new blood factor of the L system in pigs. Anim. Blood Grps Biochem. Genet. 2, 243-245.

Lopez-Corrales, N.L., Beattie, C.W., Rohrer, G.A., 1999: Cytogenetic assignment of 53 microsatellites from the USDA-MARC porcine genetic map. Cytogenet. Cell Genet. 84, 140-144.

Matousek, J., Hojny, J., Janik, A., 1986: Serum allotypes in ovarian folicular fluids of pigs. Anim. Genet. 17, 169-174.

Muladno, G., Brown, S.C., Moran, C., 1996: Microsatellite mapping of the brain natriuretic peptide (BNP1) locus to porcine chromosome 6. Anim. Genet. 27, 289.

Nielsen, P.B., Vögeli, P., 1982: A new Kd subgroup designated Kg in the porcine K blood group system. Anim. Blood Grps. Biochem. Genet. 13, 65-66.

Nielsen, V.H., Larsen, N.J., 1997: RFLPs at the porcine growth hormon releasing hormone (GHRH) gene. Anim. Genet. 28, 152.

Otsu, K., Phillips, M.S., Khanna, V.K., De Leon, S., MacLennan, D.H., 1992: Refinement of diagnostic assays for a probable causal mutation for procine and human malignant hyperthermia. Genomics 13, 835-837.

Rapacz, J., Hasler-Rapascz, J., 1982: Immunogenetic studies on polymorphism, postnatal passive acquisition and development of immunoglobulin gamma (IgG) in swine. Proceedings of the 2nd World Congress on Genetics Applied to Livestock Production 8, 601-606.

Rapacz, J., Hasler-Rapacz, J., Kuo, W.H., 1986: Immunogenetic polymorphism of lipoproteins in swine: genetic, immunological and physiochemical characterization of the two allotypes Lpr1 and Lpr2. Genetics 113, 985-1007.

Rapacz, J., Hasler-Rapacz, J.O., Hu, Z.L., Rapacz, J.M., Vögeli, P., Hojny, J., Janik, A., 1994: Identification of new apolipoprotein B epitopes and haplotypes and their distribution in swine populations. Anim. Genet. 25, Suppl. 1, 51-57.

Reiner, G., Heinricy, L., Brenig, B., Geldermann, H., Dzapo, V., 2000a: Cloning, structural organization, and chromosomal assignment of the porcine c-fos proto-oncogene, FOS. Cytogen. Cell Genet. 89, 59-61.

Reiner, G., Willems, H., Geldermann, H., Dzapo, V., 2000b: Four PCR-RFLPs and a sequence polymorphism in the porcine c-myc proto-oncogene and confirmation of the chromosomal localisation on SSC4 by linkage mapping. Anim. Genet. 31, 155-156.

Riquet, J., Milan, D., Woloszyn, N., Schmitz, A., Pitel, F., Frelat, G., Gellin, J., 1995: A linkage map with microsatellites isolated from swine flow-sorted chromosome 11. Mamm. Genome 6, 623-628.

Robic, A., Dalens, M., Woloszyn, N., Milan, D., Riquet, J., Gellin, J., 1994: Isolation of 28 new porcine microsatellites revealing polymorphism. Mamm. Genome 5, 580-583.

Robic, A., Parrou, J.L., Yerle, M., Goureau, A., Dalens, M., Milan, D., Gellin, J., 1995: Pig microsatellites isolated from cosmids revealing polymorphism and localized on chromosomes. Anim. Genet. 26, 1-6.

Robic, A., Milan, D., Woloszyn, N., Riquet, J., Yerle, M., Nagel, M., Bonnett, M., Pinton, P., Dalens, M., Gellin, J., 1997: Contribution to the physically anchored linkage map of the pig. Anim. Genet. 28, 94-102.

Rohrer, G.A., Alexander, L.J., Keele, J.W., Smith, T.P., Beattie, C.W., 1994: A microsatellite linkage map of the porcine genome. Genetics 136, 231-245.

Rohrer, G.A., Alexander, L.J., Hu, Z.L., Smith, T.P., Keele, J.W., Beattie, C.W., 1996: A comprehensive map of the porcine genome. Genome Res. 6, 371-391.

Rohrer G.A., 1999: Androgen receptor (AR) maps to Xp1.1-q1.1 in the porcine genome. J. Anim. Sci. 77, 499-500.

Russo, V., Fontanesi, L., Davoli, R., Nanni Costa, L., Cagnazzo, M., Buttazzoni, L., Virgili, R., Yerle, M., 2002: Investigation of candidate genes for meat quality in dry-cured ham production: the porcine cathepsin B (CTSB) and cytostatin B (CSTB) genes. Anim. Genet. 33, 123-131.

Ruyter, D., Verstege, A.J.M., Van der Poel, J.J., Groenen, M.A.M., 1994: Five porcine polymorphic microsatellite markers. Anim. Genet. 25, 53.

Saison, R., 1967: Two new antibodies, anti-Nb and anti-Nc in the N blood-group system in pigs. Vox Sang. 12, 215-220.

Smith, T.P.L., Rohrer, G.A., Alexander, L.J., Troyer, D.L., Kirby-Dobbels, K.R., Janzen, M.A., Cornwell, D.L., Louis, C.F., Schook, L.B., Beattie, C.W., 1995: Directed integration of the physical and genetic linkage maps of swine chromosome 7 reveals that the SLA spans the centromere. Genome Res. 5, 259-271.

Schmitz, C.B., Rothschild, M.F., Tuggle, C.K., 1993: BslI polymorphism at the swine alpha-actinin2 locus. J. Anim. Sci. 71, 3477.

Schmitz, C.B., Rothschild, M.F., Tuggle, C.K., 1994: Length polymorphism in the swine alpha-actin locus. J. Anim. Sci. 72, 1910.

Stratil, A., Čepica, S., Cizova-Schröffelova, D., Geldermann, H., 1997a: Some new variants of serum protease inhibitors in Meishan pigs. Anim. Genet. 28, 446-447.

Stratil, A., Peelman, L.J., Van Poucke, M., Čepica, S., 1997b: A HinfI PCR-RFLP at the porcine leptin (LEP) gene. Anim. Genet. 28, 371-372.

Stratil, A., Kopecny, M., Moser, G., Schroeffel, J., Čepica, S., 1998: HpaII and RsaI PCR-RFLPs within an intron of the porcine leptin receptor gene (LEPR) and its linkage mapping. Anim. Genet. 29, 405-406.

Stratil, A., Čepica, S., 1999: Three polymorphisms in the porcine myogenic factor 5 (MYF5) gene detected by PCR-RFLP. Anim. Genet. 30, 79-80.

Stratil, A., Knoll, A., Moser, G., Kopecny, M., Geldermann, H., 2000: The porcine adenosine monophosphate deaminase 1 (AMPD1) gene maps to chromosome 4. Anim. Genet. 31, 147-148.

Stratil A., Kubícková S., Peelman L.J., Reiner G., Musilová P., Van Poucke M., Rubes J., Geldermann H., 2001a: FISH, RH and linkage assignment of the porcine ABCD3 (PXMP1) gene to the distal end of chromosome 4q. Anim. Genet. 32, 323-5.

Stratil A., Reiner G., Peelman L.J., Van Poucke M., Geldermann H., 2001b: Linkage and radiation hybrid mapping of the porcine gene for subunit C of succinate dehydrogenase complex (SDHC ) to chromosome 4. Anim. Genet. 32, 110-2.

Stratil A., Kubícková S., Archibald A.L., Peelman L.J., McClenaghan M., Musilová P., Van Poucke M., Rubes J., 2002a: Assignment of the porcine GLUL gene to the distal end of chromosome 9q. Anim. Genet. 33, 315-6.

Stratil, A., Peelman, L.J., Mattheuws, M., Van Poucke, M., Reiner, G., Geldermann, H., 2002b: A novel porcine gene, α-1-antichymotrypsin 2 (SERPINA3-2): sequence, genomic organization, polymorphism and mapping. Gene 292, 113-119.

Stratil A., Reiner G., Peelman L.J., Davoli R., Van Poucke M., Zambonelli P., Geldermann H., 2002c: An Alw 26I PCR-RFLP in exon 1 of the porcine SKI oncogene and mapping the gene to the RYR1 ( CRC ) linkage group on chromosome 6. Anim. Genet. 33, 377-9.

Stratil A., Blazková P., Kopecný M., Bartenschlager H., Van Poucke M., Peelman L.J., Fontanesi L., Davoli R., Scotti E., Russo V., Geldermann H., 2003: Characterization of a SINE indel polymorphism in the porcine AGL gene and assignment of the gene to chromosome 4q. Anim. Genet. 34, 146-8.

Stratil A., Wagenknecht D., Van Poucke M., Kubícková S., Bartenschlager H., Musilová P., Rubes J., Geldermann H., Peelman L.J., 2004: Comparative and genetic analysis of the porcine glucocerebrosidase (GBA) gene. Comp. Biochem. Physiol. B Biochem. Mol. Biol. 138, 377-83.

Stratil A., Van Poucke M., Bartenschlager H., Knoll A., Yerle M., Peelman L.J., Kopecný M., Geldermann H., 2006: Porcine OGN and ASPN: mapping, polymorphisms and use for quantitative trait loci identification for growth and carcass traits in a Meishan x Piétrain intercross. Anim. Genet. 37, 415-8.

Stratil A., Knoll A., Horák P., Bílek K., Bechynová R., Bartenschlager H., Van Poucke M., Peelman L.J., Svobodová K., Geldermann H., 2008: Mapping of the porcine FBN2, YWHAQ, CNN3, DCN, POSTN, SPARC, RBM39 and GNAS genes, expressed in foetal skeletal muscles. Anim. Genet. 39, 204-5.

Tuggle, C.K., Yu, T.P., Helm, J.M., Rothschild, M.F., 1993: Cloning and restriction fragment length polymorphism analysis of a cDNA for swine PIT-1, a gene controlling growth hormone expression. Anim. Genet. 24, 17-21.

Wada Y., Akita T., Awata T., Furukawa T., Sugai N., Inage Y., Ishii K., Ito Y., Kobayashi E., Kusumoto H., Matsumoto T., Mikawa S., Miyake M., Murase A., Shimanuki S., Sugiyama T., Uchida Y., Yanai S., Yasue H., 2000: Quantitative trait loci (QTL) analysis in a Meishan x Göttingen cross population. Anim. Genet. 31, 376-84.

Wagenknecht D., Stratil A., Bartenschlager H., Van Poucke M., Peelman L.J., Majzlík I., Geldermann H., 2003: Linkage and radiation hybrid mapping of the porcine MEF2D gene to chromosome 4q. Anim. Genet. 34, 232-3.

Wagenknecht D., Bartenschlager H., Van Poucke M., Geldermann H., Peelman L.J., Majzlík I., Stratil A., 2005: Linkage and radiation hybrid mapping of the porcine MPZ gene to chromosome 4q. Anim. Genet. 36, 181-2.

Wagenknecht D., Stratil A., Bartenschlager H., Van Poucke M., Peelman L.J., Majzlík I., Geldermann H., 2006: SNP identification, linkage and radiation hybrid mapping of the porcine lamin A/C (LMNA) gene to chromosome 4q. J. Anim. Breed. Genet. 123, 280-3.

Wang Z., Rohrer G.A., Stone R., Troyer D., 2000: Isolation of thirty-one new porcine microsatellites from a microsatellite enriched microdissected chromosome 8 library. Anim. Biotechnol. 11, 33-43.

Wilke, K., Jung, M., Chen, Y., Geldermann, H., 1994: Porcine (GT)n sequences: structure and association with dispersed and tandem repeats. Genomics 21, 63-70.

Winterø, A.K., Chowdhary, B., Fredholm, M., 1994a: A porcine polymorphic microsatellite locus (S0076) at chromosome 13q12. Anim. Genet. 25, 430.

Winterø, A.K., Fredholm, M., Thomsen, P.D., 1994b: A porcine polymorphic microsatellite locus (S0077) at chromosome 16q14. Anim. Genet. 25, 122.
