# Supplementary material for: Genome-wide mapping of Quantitative Trait Loci for fatness, fat cell characteristics and fat metabolism in three porcine F2 crosses
Source: Genet Sel Evol. 2010 Jul 28;42(1):31. doi: 10.1186/1297-9686-42-31 (PMC2923101; doi:10.1186/1297-9686-42-31)
Supplement: Additional file 2 — Parameters of the traits. Numbers of observations, phenotypic means, standard deviations and determination coefficients are given for the considered traits of the F2 animals and each cross. [file 1297-9686-42-31-S2.DOCX]

**Suppl. Table 2:** Parameters of the traits

Numbers of observations, phenotypic means, standard deviations and determination coefficients from the reduced model (initial model) for the F_2_ animals of each cross and the considered traits. The initial model includes continuous (age at slaughter) and discontinuous (2-month classes as seasonal factor, sex and litter number) independent variables. For the cross WxM and the fat cell traits the factors 2-month class and litter number were excluded due to low observation numbers.

|  |  | MxP | | | |  | WxP | | | |  | WxM | | | |
| --- | --- | --- | --- | --- | --- | --- | --- | --- | --- | --- | --- | --- | --- | --- | --- |
| Trait ^a^) | Unit | N | Mean | SD | r^2^ (%) |  | N | Mean | SD | r^2^ (%) |  | N | Mean | SD | r^2^ (%) |
|  |  |  |  |  |  |  |  |  |  |  |  |  |  |  |  |
| CW | [kg] | 316 | 76.217 | 14.191 | 16.7 |  | 315 | 57.141 | 12.602 | 12.1 |  | 335 | 54.752 | 11.706 | 12.2 |
| AFW | [kg] | 316 | 0.862 | 0.410 | 19.2 |  | 315 | 0.497 | 0.254 | 17.2 |  | 335 | 0.926 | 0.382 | 11.1 |
| HEFW | [kg] | 316 | 2.599 | 0.818 | 16.9 |  | 315 | 1.421 | 0.490 | 16.2 |  | 335 | 2.264 | 0.662 | 10.9 |
| SEFW | [kg] | 316 | 1.263 | 0.355 | 21.5 |  | 315 | 0.736 | 0.233 | 16.2 |  | 335 | 1.026 | 0.294 | 13.6 |
| BFW | [kg] | 316 | 2.406 | 0.908 | 24.2 |  | 315 | 1.538 | 0.653 | 21.1 |  | 335 | 2.273 | 0.783 | 10.9 |
| FCP | [%] | 316 | 18.334 | 3.779 | 25.1 |  | 315 | 14.208 | 3.000 | 23.5 |  | 335 | 23.217 | 3.422 | 10.2 |
| BFML | [mm] | 316 | 21.957 | 6.936 | 27.0 |  | 315 | 16.763 | 5.853 | 29.6 |  | 335 | 31.620 | 8.615 | 14.6 |
| FD10 | [mm] | 316 | 23.396 | 6.210 | 19.7 |  | 315 | 20.057 | 4.819 | 17.7 |  | 335 | 26.794 | 6.519 | 9.8 |
| ABFD | [mm] | 316 | 27.928 | 6.684 | 22.5 |  | 315 | 22.825 | 4.976 | 21.3 |  | 335 | 31.822 | 6.677 | 15.3 |
| FAML | [cm^2^] | 316 | 20.852 | 5.885 | 20.5 |  | 313 | 16.710 | 5.521 | 23.5 |  | 335 | 24.418 | 6.590 | 17.4 |
| FMR |  | 316 | 0.725 | 0.217 | 23.1 |  | 313 | 0.513 | 0.150 | 30.9 |  | 335 | 1.272 | 0.351 | 17.3 |
|  |  |  |  |  |  |  |  |  |  |  |  |  |  |  |  |
| MDHO | [u/g t] | 315 | 0.536 | 0.269 | 16.1 |  | 315 | 0.371 | 0.148 | 18.2 |  | 326 | 0.437 | 0.172 | 19.1 |
| PCO | [mg/g t] | 315 | 3.590 | 1.536 | 24.1 |  | 315 | 4.865 | 1.729 | 13.5 |  | 326 | 3.491 | 1.117 | 11.8 |
| LGSEO | ^b^) | 315 | 2.975 | 0.182 | 16.7 |  | 308 | 2.805 | 0.172 | 23.5 |  | 325 | 2.911 | 0.130 | 17.6 |
| MDHI | [u/g t] | 315 | 0.679 | 0.310 | 16.1 |  | 315 | 0.534 | 0.224 | 22.5 |  | 326 | 0.582 | 0.227 | 21.5 |
| PCI | [mg/g t] | 315 | 4.106 | 2.200 | 25.4 |  | 315 | 5.676 | 2.348 | 16.0 |  | 326 | 3.564 | 1.337 | 12.3 |
| LGSEI | ^b^) | 313 | 3.070 | 0.175 | 19.1 |  | 313 | 2.938 | 0.174 | 23.1 |  | 326 | 3.017 | 0.122 | 17.8 |
| MDHOI | [u/g t] | 315 | 0.607 | 0.274 | 17.5 |  | 315 | 0.452 | 0.176 | 21.9 |  | 326 | 0.510 | 0.186 | 22.9 |
| PCOI | [mg/g t] | 315 | 3.848 | 1.783 | 27.0 |  | 315 | 5.270 | 1.955 | 14.9 |  | 326 | 3.528 | 1.164 | 13.2 |
| LGSEOI | ^b^) | 313 | 3.029 | 0.169 | 20.1 |  | 306 | 2.882 | 0.165 | 23.8 |  | 325 | 2.970 | 0.115 | 20.3 |
|  |  |  |  |  |  |  |  |  |  |  |  |  |  |  |  |
| FN73 | [%] | 307 | 8.989 | 6.480 | 11.2 |  | 296 | 15.417 | 7.116 | 16.2 |  | 91 | 5.912 | 5.099 | 3.2 |
| FN92 | [%] | 307 | 15.944 | 9.489 | 7.8 |  | 296 | 23.358 | 8.378 | 11.8 |  | 91 | 9.742 | 7.695 | 5.1 |
| FN114 | [%] | 307 | 21.440 | 10.549 | 5.4 |  | 296 | 17.636 | 10.315 | 17.4 |  | 91 | 15.337 | 9.761 | 1.0 |
| FN146 | [%] | 307 | 14.109 | 10.661 | 11.8 |  | 296 | 4.951 | 5.268 | 10.9 |  | 91 | 13.209 | 9.705 | 7.5 |
| FN185 | [%] | 307 | 2.507 | 4.311 | 11.8 |  | 296 | 0.506 | 0.633 | 3.8 |  | 91 | 2.732 | 3.492 | 5.1 |
| FNCM | [%] | 307 | 46.370 | 19.019 | 5.5 |  | 296 | 56.411 | 14.796 | 14.6 |  | 91 | 30.992 | 17.990 | 3.0 |
| FNCL | [%] | 307 | 16.857 | 13.925 | 13.2 |  | 296 | 5.574 | 5.778 | 10.3 |  | 91 | 16.156 | 12.508 | 7.6 |
| RFNCSL |  | 307 | 9.665 | 23.196 | 4.6 |  | 296 | 19.735 | 27.461 | 12.1 |  | 91 | 13.746 | 30.442 | 0.8 |
| RNFCML |  | 307 | 7.527 | 10.034 | 9.0 |  | 296 | 21.588 | 17.591 | 15.0 |  | 91 | 5.102 | 9.714 | 4.0 |
| RFNCLO |  | 307 | 17.193 | 30.054 | 5.4 |  | 296 | 41.323 | 40.926 | 13.4 |  | 91 | 18.847 | 35.041 | 1.2 |
| FV73 | [%] | 307 | 4.956 | 5.637 | 9.2 |  | 296 | 10.675 | 7.555 | 19.5 |  | 91 | 3.583 | 4.345 | 4.9 |
| FV92 | [%] | 307 | 14.549 | 10.471 | 14.5 |  | 296 | 27.350 | 10.142 | 14.1 |  | 91 | 10.182 | 9.051 | 9.2 |
| FV114 | [%] | 307 | 30.045 | 11.247 | 7.8 |  | 296 | 32.834 | 11.208 | 17.8 |  | 91 | 24.902 | 11.915 | 6.4 |
| FV146 | [%] | 307 | 34.105 | 15.507 | 10.5 |  | 296 | 17.322 | 11.952 | 15.0 |  | 91 | 38.610 | 14.814 | 8.7 |
| FV185 | [%] | 307 | 10.045 | 10.937 | 12.2 |  | 296 | 3.975 | 2.893 | 5.5 |  | 91 | 14.234 | 12.119 | 3.6 |
| FVCM | [%] | 307 | 49.550 | 20.777 | 13.6 |  | 296 | 70.859 | 12.425 | 11.9 |  | 91 | 38.668 | 20.608 | 10.0 |
| FVCL | [%] | 307 | 46.697 | 22.414 | 13.6 |  | 296 | 23.562 | 13.119 | 12.6 |  | 91 | 56.087 | 21.935 | 9.0 |
| RFVCSL |  | 307 | 0.157 | 0.333 | 4.8 |  | 296 | 0.351 | 0.403 | 13.3 |  | 91 | 0.162 | 0.325 | 1.9 |
| RFVCML |  | 307 | 1.739 | 1.695 | 11.9 |  | 296 | 4.183 | 2.655 | 14.2 |  | 91 | 1.198 | 1.677 | 5.8 |
| RFVCLO |  | 307 | 1.896 | 1.914 | 11.1 |  | 296 | 4.534 | 2.870 | 14.1 |  | 91 | 1.361 | 1.917 | 5.4 |

F_2_ crosses: MxP, Meishan ♂ x Pietrain ♀; WxP, European Wild Boar ♂ x Pietrain ♀; WxM, European Wild Boar ♂ x Meishan ♀.

^a^) For definition of trait acronyms see **Table 2**.

^b^) [log_10_(units/g tissue * 1000)]
