# Supplementary material for: Genome-wide mapping of Quantitative Trait Loci for fatness, fat cell characteristics and fat metabolism in three porcine F2 crosses
Source: Genet Sel Evol. 2010 Jul 28;42(1):31. doi: 10.1186/1297-9686-42-31 (PMC2923101; doi:10.1186/1297-9686-42-31)
Supplement: Additional file 3 — Genome-wide threshold values. The threshold values, which were calculated according to [19] and with 1000 permutations, are listed for the P < 0.05 and P < 0.01 significance levels. [file 1297-9686-42-31-S3.DOCX]

**Suppl. Table 3** Genome-wide threshold values

The threshold values were calculated according to [19] using 1000 permutations and the averaged results of six traits (SEFW, FD10, MDHOI, PCOI, FNCM, FVCL; for trait acronyms see **Table 2**) for all crosses.

| F_2_ animals | Genetic components | Significance level | Threshold values | | VF_2_ [%] |
| --- | --- | --- | --- | --- | --- |
|  |  |  | *F* ratio | P |  |
| Male and female | Additive and dominant | 0.01 | 10.56 | .000132 | 5.92 |
|  |  | 0.05 | 8.47 | .000545 | 4.69 |
| Male and female | Additive | 0.01 | 16.99 | .000055 | 4.83 |
|  |  | 0.05 | 13.42 | .000297 | 3.79 |
| Female | Additive and dominant | 0.01 | 11.06 | .000079 | 12.14 |
|  |  | 0.05 | 8.83 | .000516 | 9.70 |
| Male, SSCX | Additive | 0.01 | 17.03 | .000066 | 9.15 |
|  |  | 0.05 | 13.48 | .000336 | 7.28 |

*F* ratio: Percentage of F_2_ phenotypic variance explained by the QTL; VF_2_: Proportion of error variance reduction by inclusion of genetic components in the initial model.
